# The chromosome-based lavender genome provides new insights into Lamiaceae evolution and terpenoid biosynthesis

**DOI:** 10.1038/s41438-021-00490-6

**Published:** 2021-03-01

**Authors:** Jingrui Li, Yiming Wang, Yanmei Dong, Wenying Zhang, Di Wang, Hongtong Bai, Kui Li, Hui Li, Lei Shi

**Affiliations:** 1 Key Laboratory of Plant Resources and Beijing Botanical Garden, Institute of Botany, Chinese Academy of Sciences, Xiangshan 100093 Beijing, China; 2 University of Chinese Academy of Sciences, 100015 Beijing, China; 3 Novogene Bioinformatics Institute, 100083 Beijing, China

**Keywords:** Genome, Plant evolution

## Abstract

The aromatic shrub *Lavandula angustifolia* produces various volatile terpenoids that serve as resources for essential oils and function in plant-insect communication. To better understand the genetic basis of the terpenoid diversity in lavender, we present a high-quality reference genome for the Chinese lavender cultivar ‘Jingxun 2’ using PacBio and Hi-C technologies to anchor the 894.50 Mb genome assembly into 27 pseudochromosomes. In addition to the γ triplication event, lavender underwent two rounds of whole-genome duplication (WGD) during the Eocene–Oligocene (29.6 MYA) and Miocene–Pliocene (6.9 MYA) transitions. As a result of tandem duplications and lineage-specific WGDs, gene families related to terpenoid biosynthesis in lavender are substantially expanded compared to those of five other species in Lamiaceae. Many terpenoid biosynthesis transcripts are abundant in glandular trichomes. We further integrated the contents of ecologically functional terpenoids and coexpressed terpenoid biosynthetic genes to construct terpenoid-gene networks. Typical gene clusters, including *TPS-TPS*, *TPS-CYP450*, and *TPS-BAHD*, linked with compounds that primarily function as attractants or repellents, were identified by their similar patterns of change during flower development or in response to methyl jasmonate. Comprehensive analysis of the genetic basis of the production of volatiles in lavender could serve as a foundation for future research into lavender evolution, phytochemistry, and ecology.

## Graphical abstract

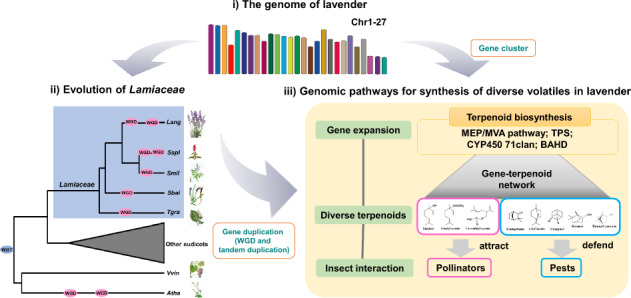

## Introduction

*Lavandula* is a distinctive genus that belongs to the species-rich and chemically diverse subclade Nepetoideae (3600 species) within Lamiaceae (~7173 species). The genus *Lavandula* consists of 39 species and has over 2500 years of recorded use by humans as flavor additives, fragrances, alternative medicine, and anti-herbivore agents^1,2^. Lavender grows spontaneously in the Mediterranean region, and ‘Jingxun 2’ is a new lavender cultivar recently bred by Chinese researchers and is characterized by high levels of linalyl acetate and linalool and low amounts of camphor in its essential oil (EO), making it the most valued lavender oil additive to many over-the-counter complementary medicines and cosmetic products in China.

Spatially restricted and structurally diverse specialized metabolites are generally used by plants as a chemical language for their interactions with the environment, such as in herbivore deterrence or pollinator attraction^3,4^. Insect pollinators play an important role in lavender reproduction as they carry out cross-pollination. Mono- and sesquiterpenoids are the main compounds in lavender essential oil (EO) and are primarily stored in an epidermal secretory structure, namely, the glandular trichome (GT)^5,6^. As phytochemical diversity in the GT drives the diversity of the lavender-visiting insect community, intraspecific gene flow and chemical defense compound levels are elevated, giving rise to genetic diversity and adaptation to hostile environments^1,5,7^.

Genetic and biochemical studies have highlighted that the locations of biosynthetic genes for specialized chemicals are not randomly scattered but adjacent to one another in the genome, that is, in metabolic gene clusters^4,8^. Terpene synthases (TPSs) are crucial for terpenoid structural diversity and generate diverse scaffolds and various tailoring enzymes, including cytochrome P450 (CYP450), acyltransferases, 2-oxoglutarate-dependent dioxygenases, methyltransferases, and glycosyltransferases, which modify and further diversify scaffolds^9–13^. Physical connections of these gene families are found in the biosynthesis of specialized terpenoids, which are often important as medicines, fragrances, and insecticides^8,14–16^.

Gene duplication and neofunctionalization appear to drive the evolution of plant gene clusters, resulting in the diversification of specialized terpenoids^15,17^. Duplicated genes are prevalent in plant genomes and have arisen primarily via whole-genome duplications (WGDs; polyploidy) and tandem duplications (TD)^18^. The expansion of the *TPS*, *CYP450*, and *BAHD* gene families in several plants often occurs via these two routes^9,19–21^. These duplicates provide the available materials that contribute to the evolution of divergent functions, such as inducing resistance to pests, increasing tolerance to stress, and promoting plants to gain some adaptive advantages, thus leading to the enhancement of plant fitness^18,22^. Clustering of terpenoid biosynthetic genes is likely to facilitate the coinheritance and coexpression of beneficial gene combinations, thus potentially reducing the possibility of the loss of single pathway genes following recombination events and incomplete cluster expression^23,24^. Based on transcriptome analysis, recent investigations have indicated that ancient polyploidization events have occurred in Lamiaceae. Moreover, frequent WGDs predicted within Nepetoideae may correlate with the species richness of this subclade^17,25–30^. The expansion of terpenoid biosynthetic gene families is closely correlated with mono- and sesquiterpenoid diversity. For example, the expansion of the TPS-a subfamily is responsible for the synthesis of sesquiterpenes in patchouli, and the size of the TPS-b subfamily has a positive relationship with monoterpene diversity^28^.

As a representative volatile renewable resource, *Lavandula angustifolia* has the potential to be developed as a genomic model for studying terpenoid production. Although a draft genome of lavender has been generated from second-generation sequencing results^31^, the vast majority of the lavender metabolic gene clusters and the evolution of duplicated genes remain undiscovered. Here, we provide a chromosome-level genome of lavender. Furthermore, our work revealed that WGD and TD promoted diversification and terpenoid variation within the Lamiaceae, and the resulting expansive gene families were correlated with lavender adaption to the Mediterranean environment. Meanwhile, gene-terpenoid networks indicated the combinational function of physically linked and co-expressed gene clusters related to specific terpenoid biosynthesis. Our study could serve as a foundation for future research into Lamiaceae evolution, phytochemistry, and ecology.

## Results

### Chromosome-level assembly of the lavender genome

**Fig. 1:**
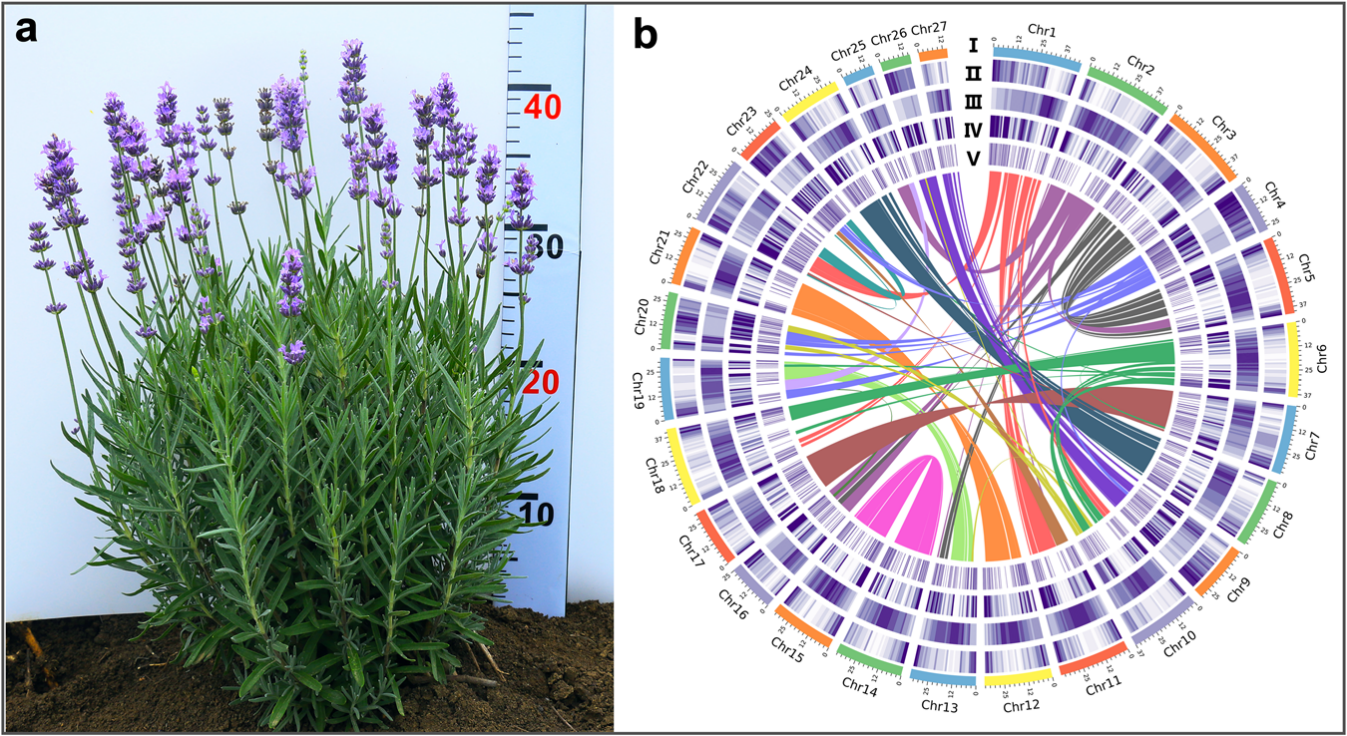
Genomic landscape of lavender. **a** Photo of *L. angustifolia* ‘Jingxun 2’ cultivated in Beijing. **b** Lavender genomic landscape. (I) Circular representation of the pseudomolecule. (II–IV) Gene density (500kb window), percent repeats (500kb window), and ncRNA content (500kb window). (V) Locations of tandem duplicated genes. Each line in the center of the circle connects a pair of homologous genes

Based on k-mer distribution analysis, *L. angustifolia* ‘Jingxun 2’ was estimated to have a genome size of 1094.97 Mb (Supplementary Fig. S1), which was close to the genome size of ~1016.25 Mb determined experimentally by flow cytometry (Supplementary Fig. S2). K-mer analysis with a length of 17 indicated that the genome had high heterozygosity (0.76%) and a high repetitive sequence content (69.23%) (Supplementary Tables S1 and S2); thus, complementary approaches were combined to obtain the lavender reference genome assembly (Supplementary Fig. S3). We generated 112.15 Gb of PacBio RSII reads, affording ~103-fold coverage of the lavender genome. Following interactive error correction of the PacBio reads, the assembly was carried out using FALCON to obtain primary contigs. The final contigs were error corrected with 59.06 Gb of short reads obtained from the Illumina NovaSeq 6000 sequencer. Consensus sequences were further assembled with the assistance of 117.77 Gb (108.05 coverage) clean data produced from the 10× Genomics library (Supplementary Table S3). All contigs were extended using FragScaff to generate an assembly with a total contig length of 911.14 Mb (90% of the genome) and an N50 of 1.20 Mb (Fig. 1 and Table 1). To confirm the completeness of the genome assembly, we used Illumina short reads and performed CEGMA and BUSCO assessments. Our results showed that more than 98.2% of Illumina short reads could be mapped to the genome (Supplementary Table S4), 98.79% of genes could match 248 core eukaryotic genes (CEGs), and 91.4% of complete BUSCOs were assembled (Table 1 and Supplementary Tables S5, S6), indicating a high degree of completeness of the lavender genome assembly.

**Table 1:** Major indicators of the *L. angustifolia* genome.

Parameter	*L. angustifolia*
Estimate of genome size	1016.25 M
Estimate of genome size (survey)	1094.97 M
Total length of contigs	911.14 M
Total number of contigs	1383
N50 of contigs (bp)	1.22 M
Largest contig (bp)	9,968,313
Total length of scaffolds	914.49 M
Total number of scaffolds	306
N50 of scaffolds (bp)	36.20 M
Largest scaffold (bp)	46,645,376
GC content	38.58%
Complete BUSCOs	91.4%
Repeat density	58.28%
Number of protein-coding genes	65,905
Average length of transcripts (bp)	2739
Average length of coding-sequences (bp)	1114
Number of annotated genes	62,822
Number of miRNA	1351
Number of tRNA	1298
Number of rRNA	399
Number of snRNA	1199

Moreover, contigs were corrected and scaffolded by high-throughput chromatin conformation capture (Hi-C) into pseudochromosomes. Of the 914.49 Mb of scaffold sequences, 97.82% of bases were anchored to 27 superscaffolds (pseudochromosomes), which corresponded well to the number of chromosomes identified by our experiment (1*n* = 27, 2*n* = 54) (Supplementary Tables S7, S8 and Supplementary Figs. S4, S5). The final chromosome-level genome assembly of *L. angustifolia* was 894.50 Mb with an N50 of 36.2 Mb, a GC content of 38.58%, and only 0.37% N (Table 1 and Supplementary Table S9).

### Genome annotation and phylogeny of lavender

To obtain a reliable gene structure, we generated a full-length transcriptome and RNA-Seq data of various tissues to facilitate the accuracy of genome annotation (Supplementary Tables S10, S11 and Supplementary Fig. S6). We found that 58.28% (575.49 Mb) of the lavender genome is composed of repetitive sequences (Table 1 and Supplementary Table S12), and long terminal repeats are the predominant transposable elements, accounting for 51.94% of the whole genome (Supplementary Table S13 and Supplementary Fig. S7). A high-confidence set of 65,905 protein-coding genes was predicted, of which 95.30% (62,822) had homologs annotated in public protein databases and 91.3% could be located on the 27 pseudochromosomes (Table 1 and Supplementary Tables S14–S16). We also identified non-coding RNAs (representing 6.67% of the genome), including 1351 miRNAs, 1298 transfer RNAs, 1199 small nuclear RNAs, and 399 ribosomal RNAs (Table 1 and Supplementary Table S17).

**Fig. 2:**
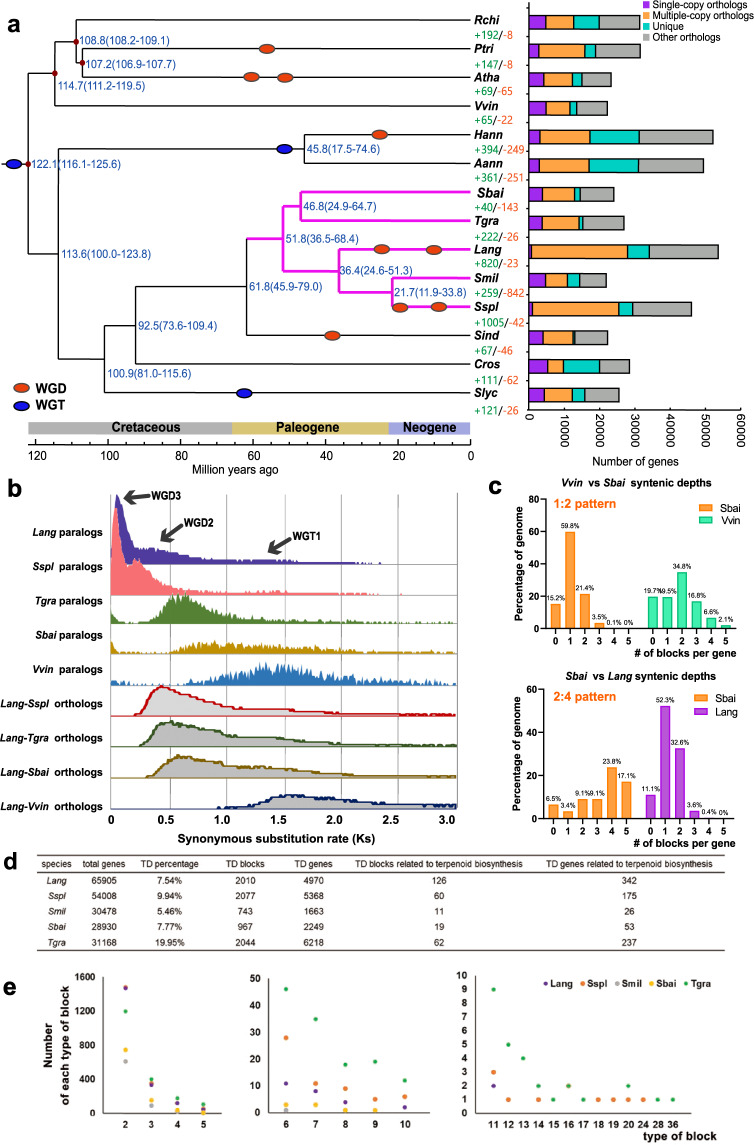
Lavender genome evolution. **a** Phylogenetic tree with 59 single-copy orthologs from 13 species identified by OrthoMCL to show divergence times. The distribution of genes in each species is shown in the right panel. **b** Synonymous substitution rate (Ks) distributions of syntenic blocks for lavender paralogs and orthologs with *Vvin* and some species (*Smil*, *Sspl*, *Sbai* and *Tgra*) in Lamiaceae. *Lang*, *L. angustifolia*; *Smil*, *Salvia miltiorrhiza*; *Sspl*, *Salvia splendens*; *Slyc*, *Solanum lycopersicum*; *Sbai*, *Scutellaria baicalensis*; *Tgra*, *Tectona grandis*; *Sind*, *Sesamum indicum*; *Cros*, *Catharanthus roseus*; *Rchi*, *Rosa chinensis*; *Hann*, *Helianthus annuus*; *Aann*, *Artemisia annua*; *Ptri*, *Populus trichocarpa*, *Vvin*, *Vitis vinifera*; *Atha*, *Arabidopsis thaliana*. **c** The syntenic blocks among *Lang*, *Vvin*, and *Sbai*. **d** Summary of tandem duplications in lavender and four other Lamiaceae species. **e** The number of each type of tandem block in lavender and four other Lamiaceae species

A total of 17,057 orthologous protein groups encompassing 53,725 genes were found for lavender and four other species belonging to Lamiaceae [*Salvia miltiorrhiza* (*Smil*), *Scutellaria baicalensis* (*Sbai*), *Tectona grandis* (*Tgra*), and *Salvia splendens* (*Sspl*)], and nine other sequenced plant species belonging to the eudicot clade [*Solanum lycopersicum* (*Slyc*), *Catharanthus roseus* (*Cros*), *Sesamum indicum* (*Sind*), *Rosa chinensis* (*Rchi*), *Helianthus annuus* (*Hann*), *Artemisia annua* (*Aann*), *Populus trichocarpa* (*Ptri*), *Vitis vinifera* (*Vvin*), and *Arabidopsis thaliana* (*Atha*)] (Fig. 2a). Lavender genes were classified as 738 single-copy, 27,273 multiple-copy, 6182 unique, and 19,532 other genes (Fig. 2a). The sequences of the 59 single-copy orthologous genes shared by the 14 species were retrieved, and a phylogenetic tree was constructed based on these sequences. The results showed that lavender and its close relatives in Lamiaceae (*Smil*, *Sspl*, *Sbai*, and *Tgra*) clustered into one monophyletic group. These five species of Lamiaceae shared 10,122 gene families, and 2522 gene families appeared to be unique to lavender (Supplementary Fig. S8). By calibrating the divergence time based on four known species with fossils, we inferred that the *L. angustifolia* lineage seems to have diverged from the *Salvia* clade (*Smil* and *Sspl*) 36.4 (14.6–51.3) million years ago (MYA) (Fig. 2a). Notably, lavender and *Sspl* had more expanded gene families (820 for lavender and 1005 for *Sspl*) than contracted ones (42 for lavender and 23 for *Sspl*). In contrast, *Smil* had more contracted gene families (842) than expanded ones (259) (Fig. 2a). Functional analysis showed that genes among expanded and unique families of genes in lavender were preferentially enriched in the terms terpene synthase, plant–pathogen interaction, and plant hormone signal transduction (Supplementary Figs. S9 and S10).

### Lineage-specific whole-genome duplication and tandem duplication of lavender

Self-alignment of the lavender genome identified 55,696 paralogous gene pairs into 2603 syntenic blocks (Fig. 1b). Of these intragenomic syntenic blocks, 72.3% were found to be syntenic to more than one location in the genome, suggesting that more than one WGD occurred in the evolutionary history of lavender. To investigate lineage-specific WGDs in lavender, synonymous substitutions per synonymous site (Ks) were characterized in the *Sbai*, *Sspl*, *Tgra*, *Vvin*, and lavender genomes (Fig. 2b). The Ks distribution between syntenic genes of *L. angustifolia* exhibited two distinct peaks (Ks = 0.09, Ks = 0.39) in addition to a peak shared by all the eudicots, indicating two recent WGD events undergone by *L. angustifolia* after the core eudicot γ triplication event. *Sspl* also showed two signature peaks (Ks = 0.08, Ks = 0.24) in its paralogous Ks distributions, while only one paralogous peak was observed in the genomes of *Sbai* and *Tgra* (0.63 for *Sbai* and 0.68 for *Tgra*), which diverged from *L. angustifolia* after the γ event (Fig. 2b). These data revealed that the *L. angustifolia* and *Sspl* lineages shared the same WGD history and experienced additional WGD after splitting from *Sbai* and *Tgra*. These results were also verified by fourfold synonymous third-codon transversion position (4dTV) analysis (Supplementary Fig. S11). Visualization of microsynteny and statistics of syntenic blocks showed that there were approximately two copies of each syntenic block from *Vvin* in *Sbai* and four copies of two syntenic blocks from *Sbai* in *Lang* (Fig. 2c and Supplementary Fig. S12), indicating that *Sbai* and *Lang* had one and two rounds of WGD since the γ event, respectively, which is consistent with the results of Ks and 4dTV analysis. The dot plot-based investigation of paralogous blocks in lavender provides additional and clear proof of the presence of WGD polyploidization events (Supplementary Fig. S13). Using divergence time and the mean Ks values of syntenic blocks between *L. angustifolia* and *Sspl*, we estimated the synonymous substitutions per site per year as 6.59 × 10^−9^, which led to the dating of the estimated times of the two recent WGD events in *L. angustifolia* at ~29.6 and 6.86 MYA. Moreover, two additional WGDs of *Sspl* were estimated at 19.29 and 6.43 MYA.

To gain insight into the functions of retained genes following two individual WGDs, we determined whether there was an enrichment of specific molecular functions from KEGG pathways for genes with Ks values in the regions associated with the two WGDs (Supplementary Tables S18 and S19). A total of 15,234 (23.1% of the total genes) genes were retained in the penultimate WGD event, whereas 21,386 genes (32.5% of the total genes) were retained in the last WGD event. Several gene copies were associated with terpenoid metabolism and plant defense, which mapped the pathways of “terpenoid backbone biosynthesis”, “limonene and pinene degradation”, “plant hormone signal transduction”, “phenylalanine, tyrosine, and tryptophan biosynthesis”, and “brassinosteroid biosynthesis”, all of which were retained after the last WGD event (Supplementary Tables S18 and S19). These results suggest that gene-related terpenoid metabolism and regulation are crucial for lavender survival.

TD can generate a copy of several genes within the same scaffold or chromosome; these events often include genes from the same networks or pathways and can accelerate the divergence of gene function^18^. We found a total of 2010 tandem blocks including 4970 genes (7.5% of all genes) in the lavender genome and 126 tandem blocks composed of 342 genes that were associated with terpenoid biosynthesis (Fig. 2d and Supplementary Table S21). The two largest tandem blocks observed in the lavender genome were a group of 19 genes encoding the auxin-responsive protein SAUR, and a group of 14 genes encoding the pathogenesis-related protein Bet V1 (Fig. 2e and Supplementary Table S20). To investigate whether these tandemly duplicated gene families were specific to lavender, tandemly duplicated genes were investigated in other Lamiaceae species. Comparatively fewer tandem blocks were identified in *Smil* (743) and *Sbai* (967), whereas the genomes of *Sspl* and *Tgra* included 2077 and 2044 tandem blocks, respectively (Fig. 2d and Supplementary Table S21). Regarding terpenoid biosynthesis, tandem blocks and genes were less abundant in these four species than in lavender (Fig. 2d). The functions of the largest tandem gene group were different between each genome; for example, the nine UDP-glycosyltransferase genes grouped in *Sbai* and 24 genes encoding the berberine bridge enzyme were clustered in *Sspl* (Fig. 2e and Supplementary Table S21).

### Identification of lavender volatile terpenoids and their ecological function

**Fig. 3:**
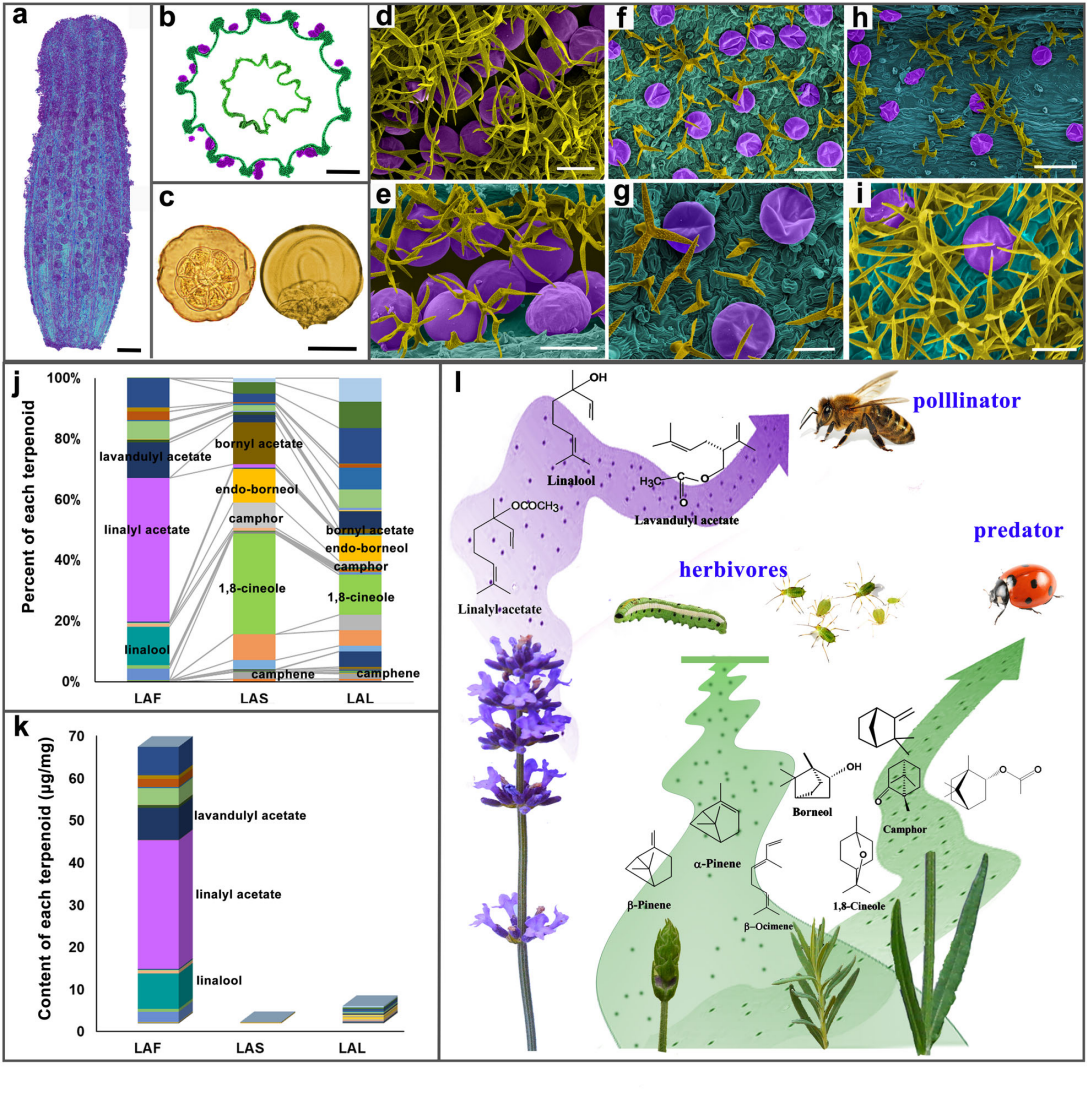
The sites, types, contents, and putative functions of volatile production in lavender. **a**, **b** Surface and cross-section of the calyx of a blossom floret. These images were captured by CT. The glandular trichomes (GTs) of lavender are colored purple. **c** Top view and side view of a single GT separated from a flower at blossom. The GTs are composed of eight secretory cells and one secretory cavity. **d**–**i** Scanning electron microscopy images. The GTs of the flower (LAF), leaf (LAL), and stem (LAS) are colored purple, and non-GTs are in yellow. Scale bars = 1 mm (**a**, **b**); 50 μm (**c**–**e**, **g**, **i**); and 100 μm (**f**, **h**). **j**, **k** The relative and absolute contents of volatile terpenoids in LAF, LAL, and LAS. **l** The ecological function of the main volatiles emitted by opening flowers, flower buds, leaves, and stems. A large proportion of linalool, linalyl acetate, and lavandulyl acetate in opening flowers function as attractants for pollinators. At the flower bud stage, *α*-pinene, *β*-pinene, and *β*-ocimene, etc. provide defense against herbivores and predators. Borneol, camphor, 1,8-cineole, camphene, and bornyl acetate are the main compounds in leaves and stems and are always repellents to pests.

Abundant GTs (Fig. 3a–e) are present on the surfaces of lavender flowers (Fig. 3a–e), leaves (Fig. 3f, g, i), and stems (Fig. 3h). Terpenoid levels often fluctuate among different organs and during various developmental stages. The contents of volatile terpenoids in the leaves, stems, and flowers of lavender were determined using SPME coupled to GC-MS (Fig. 3j, k and Supplementary Fig. S14). As the primary organ of EO production, the flower exhibited abundant GTs, especially in the veins of the calyx (Fig. 3a–e). Our study was the first to accurately determine the contents of volatile terpenoids in lavender. Here, 65.55 µg/mg was found in flowers, which was ~16-fold and ~205-fold greater than that in leaves and stems, respectively (Fig. 3k). Linalool, linalyl acetate, and lavandulyl acetate, which have sweet floral and refreshing odors that function primarily as attractants for pollinators, constitute 71.78% of the total lavender flower volatiles, with only 3.74% in the stem and 5.98% in the leaf (Fig. 3j, l and Supplementary Table S22). In contrast, borneol, camphor, 1,8-cineole, camphene, and bornyl acetate have a slightly spicy smell, exhibiting higher activity in deterring aggressive insects^32^. The above five defensive compounds make up 68.65% and 26.37% of volatiles in the stem and leaf, respectively, and only 3.12% of flower volatiles. Other terpenoids, such as *α*-pinene, *β*-ocimene, germacrene D, (E)-*β*-farnesene, and limonene, are common plant “cry for help” compounds, usually a part of herbivore-induced volatile blends (Fig. 3l).

### Evolution of genes related to terpenoid biosynthesis

**Fig. 4:**
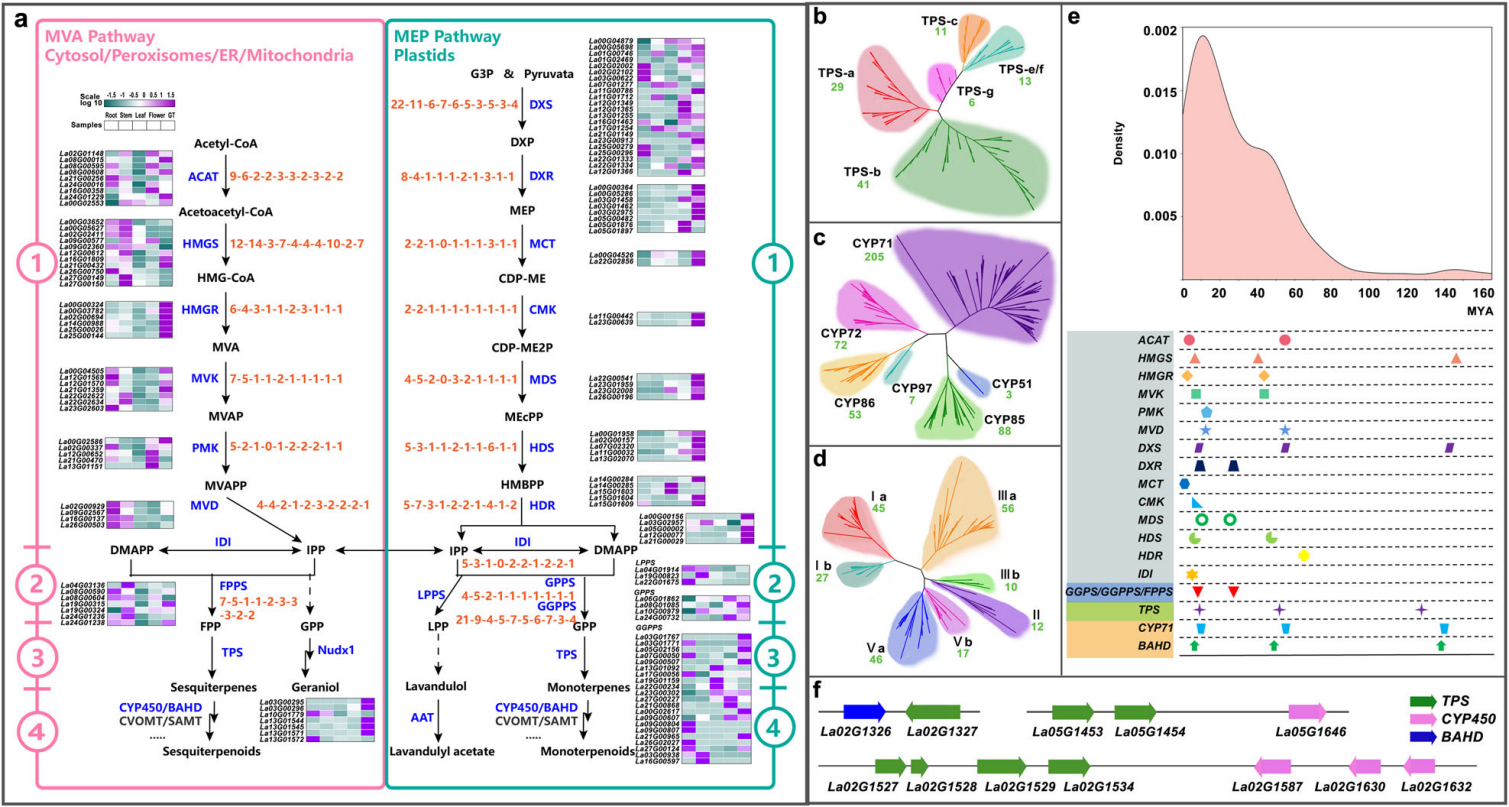
Biosynthesis of volatile terpenoid in lavender. **a** There are four steps required to produce diverse terpenoids. Enzymes involved at each step of the volatile terpenoid biosynthesis pathway are shown in blue, and intermediates are shown in black. Relative expression profiles of genes implicated in volatile terpenoid biosynthesis among various tissues (LAR, root; LAS, stem; LAL, leaf; LAF, flower; LAGT, glandular trichome) are presented as heatmaps (cyan–purple scale). Copy number variations of genes involved in volatile terpenoid biosynthesis in the ten plant species (from left to right: *Lang*, *Sspl*, *Tgra*, *Smil*, *Sbai*, *Sind*, *Slyc*, *Hann*, *Rchi*, and *Atha*) are shown in orange font. **b**–**d** Phylogeny of TPS subfamilies (**b**), CYP450 clans (**c**), and BAHD subfamilies (**d**) in lavender based on protein sequences. The gene numbers clustered into one category are indicated in green font. **e** Ks values and duplication/divergence times of genes involved in terpenoid biosynthesis in lavender. **f** Representative gene cluster with a physical link. Clusters *TPS*-*TPS*, *TPS*-*BAHD*, and *TPS*-*CYP450* are shown.

The biosynthetic pathway of terpenoids in EOs is derived from the two 5-carbon precursors “building blocks” [isopentenyl diphosphate (IPP) and dimethylallyl diphosphate (DMAPP)] generated by both the 2-C-methyl-d-erythritol-4-phosphate (MEP) and mevalonate (MVA) pathways^11,13^. A diverse array of genes expressed in this first stage, including members of 14 gene families, i.e., acyl-coenzyme A-cholesterol acyltransferase (ACAT), hydroxymethylglutaryl coenzyme A synthase (HMGS), hydroxymethylglutaryl coenzyme A reductase (HMGR), mevalonate kinase (MVK), phospho-mevalonate kinase (PMK), mevalonate diphosphate decarboxylase (MVD), 1-deoxy-d-xylulose 5-phosphate synthase (DXS), 1-deoxy-d-xylulose 5-phosphate reductoisomerase (DXR), 2-C-methyl-d-erythritol-4-phosphate cytidylyltransferase (MCT), 4-(cytidine-5-diphospho)-2-C-methyl-d-erythritol kinase (CMK), 2-C-methyl-d-erythritol-2,4-cyclodiphosphate synthase (MDS), (E)-4-hydroxy-3-methyl-but-2-enyl-pyrophosphate synthase (HDS), (E)-4-hydroxy-3-methyl-but-2-enyl-pyrophosphate reductase (HDR) and isopentenyl diphosphate isomerase (IDI), were identified in the genome of lavender and in the other nine reported plant species (*Sspl*, *Sbai*, *Smil*, and *Tgra* of Lamiaceae, *Slyc*, *Sind*, *Hann*, *Atha*, and *Rchi*). Our results showed that the copy number of those genes was expanded in lavender, especially *DXS*, which encodes the crucial rate-limiting enzyme of the MEP pathway (Fig. 4a and Supplementary Table S23). Multiple copies of genes in lavender and *Sspl* corresponding to one copy of *Smil*, *Sbai*, or *Tgra* were observed by phylogenetic analysis (Supplementary Fig. S15). Expression profiles of these genes among various tissues showed that transcripts of most gene copies associated with the first stage of terpenoid biosynthesis, such as *HMGR*, *DXS*, *DXR*, *MCT*, *CMK*, *MDS*, and *HDS*, were abundant in LAGT, where EOs are biosynthesized (Fig. 4a).

In the second stage, IPP and DMAPP are canonically condensed head-to-tail by trans-prenyltransferases to generate geranyl diphosphate (GPP) and farnesyl diphosphate (FPP). There is an irregular non-head-to-tail prenyltransferase, namely, lavandulyl diphosphate synthase (LPPS), that can generate LPP, the precursor of lavandulol^33^. As expected, the copy numbers of *GPPS*, *GGPPS*, and *FPPS* increased in lavender (Supplementary Fig. S16).

Third, TPSs were used to catalyze GPP and FPP to form the basic skeleton of monoterpenes (C10) and sesquiterpenes (C15), respectively. We identified 100 genes encoding TPSs that were classified into five subclades, excluding TPS-d and TPS-h (Fig. 4b). Evolution analysis of the TPS gene family from 10 plant species showed that the TPS-b gene clade is greatly expanded in *Lavandula* (Supplementary Table S24 and Supplementary Fig. S17). The TPS-b clade is composed mostly of monoterpene synthases, and most genes classified in the subfamily exhibited a high expression level in LAGT (Supplementary Fig. S17).

Finally, the above mono- and sesquiterpenes are often modified by CYP450 to hydroxylated products and a family of plant acyltransferases called BAHD to esters. We identified 207 members that belonged to the CYP71 clan (often hydroxylated mono- and sesquiterpenes) among a total of 428 CYP450s (Fig. 4c), and 213 members from the BAHD family were classified into Ia, Ib, II, IIIa, IIIb, Va, and Vb (Fig. 4d and Supplementary Figs. S18, S19).

We calculated the Ks for each duplicated gene pair involved in terpenoid biosynthesis and found that more duplications were generated by the recent WGD event than by the penultimate WGD event (Fig. 4e), suggesting that the recent WGD event was important to the evolution of terpenoid biosynthesis in lavender.

Genes encoding specialized metabolic pathways are physically clustered in plant genomes and often coinduced in several cases^4,23^. The genome assembly allowed us to locate all the characterized terpenoid biosynthesis genes in lavender. We found that these genes are not uniformly distributed throughout chromosomes in lavender (Supplementary Fig. S20). There are 17 concentrated blocks in Chr1, 2, 4–6, 10, 12–14, 17, 19, 21, 22, 24, and 25 assigned *TPSs* and *CYP450s*/*BAHDs*. For example, *TPS-BAHD* (*La02G01324*/*La02G01326*/*La02G01327*) and *TPS-CYP450* (*La02G01527*/*La02G01528*/*La02G01529*/*La02G01543*/*La02G01587*/*La02G01630*/*La02G01632*) gene clusters were ordered on Chr2. In addition, we found that some *TPSs* were locally duplicated, such as the above *La02G01527*/*La02G01528*/*La02G01529* at Chr2 and *La05G01453*/*La05G01454* at Chr5 (Supplementary Table S25 and Fig. 4f). We also performed a genome-wide investigation of metabolic gene clusters involved in terpenoid production in lavender using the plantiSMASH genome mining algorithm. A total of 1181 metabolic gene clusters, 34 of which are potentially involved in terpenoid biosynthesis, were identified in our study (Supplementary Fig. S20).

### Gene-terpenoid networks

**Fig. 5:**
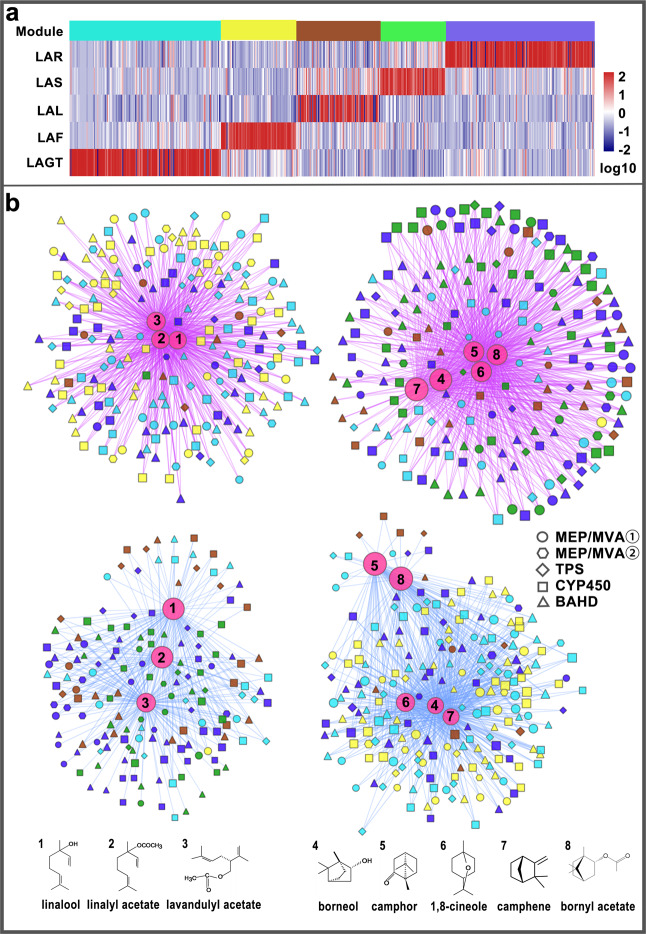
Gene-terpenoid network of candidates strongly associated with attractive and defensive terpenoids in lavender. **a** Coexpression modules of terpenoid biosynthetic genes clustered by WGCNA. **b** Candidates strongly associated with attractive and defensive terpenoids in lavender. The large circles indicate the main terpenoids in lavender. Linalool, linalyl acetate, and lavandulyl acetate have sweet floral and refreshing odors and mainly function as attractants for pollinators; borneol, camphor, 1,8-cineole, camphene, and bornyl acetate are always repellents to pests. Genes clustered into different modules are filled in green, blue, turquoise, yellow, and brown. Circles and hexagons represent genes involved in one and two steps of terpenoid biosynthesis. *TPSs*, *BAHDs*, and genes belonging to the CYP71 clan are indicated by diamonds, triangles, and squares, respectively. Edges represent the correlation between terpenoids and genes. Purple lines indicate a positive correlation, whereas cyan lines indicate a negative correlation

According to the expression profile of genes related to terpenoid biosynthesis, all these genes were grouped into five coexpression modules (Fig. 5a and Supplementary Fig. S22). Genes classified in each module exhibited similar expression patterns, presenting an organ-specific trend: blue for root, brown for leaf, green for stem, yellow for flower, and turquoise for GT.

**Fig. 6:**
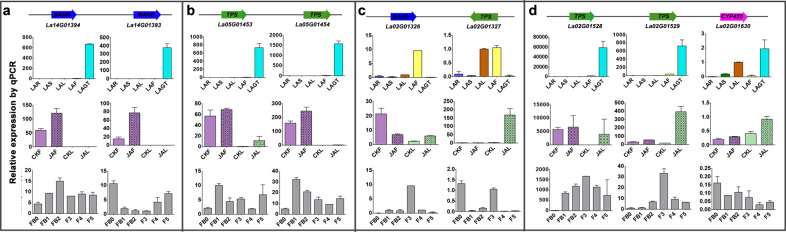
Transcriptional changes in representative genes in clusters in response to methyl jasmonate or during flower development. Gene expression patterns among various tissues (LAR, LAS, LAL, LAF, and LAGT) were verified by qRT-PCR. Relative expression levels of these genes after leaf or flower treatment with methyl jasmonate (CKL, JAL, CKF, and JAF) and in flowers at different developmental stages (FB0, FB1, FB2, F3, F4, and F5) were determined. Values shown are mean ± SE of three replicates

To assess the regulatory processes that control the accumulation of terpenoids in different organs, we searched for correlations between terpenoids and gene transcript levels, paying special attention to terpenoid biosynthetic genes. The Pearson correlation coefficient between each set of variables (metabolite or gene) was calculated. The correlation network was analyzed by Cytoscape (version 3.6.1) using a correlation coefficient > 0.7 as the cutoff. We found that most genes that showed a positive correlation with linalool, linalyl acetate, and lavandulyl acetate, the primary metabolites accumulating in flowers, were preferentially expressed in flowers and GTs. In contrast, a negative relationship between these genes and five defensive terpenoids (borneol, camphor, 1,8-cineole, camphene, and bornyl acetate) was observed (Supplementary Table S26 and Fig. 5b). The utility of the gene-to-metabolite network was also verified by observation that the expression level of key genes *La14G01394* (namely, *LaAAT*) from the turquoise module, homologs of *AAT* cloned in *L*. × *intermedia*^34^, were strongly associated with the content of lavandulyl acetate (Fig. 6a). Moreover, the expression level of *LaAAT* increased and decreased in early and late flower development, respectively, consistent with the accumulation of lavandulyl acetate (Fig. 6a). Furthermore, the transcript levels of typically clustered genes among various tissues were verified by qRT-PCR, exactly coinciding with the coexpression module in which they were grouped (Fig. 6). In addition, the expression of *TPS-TPS*, *TPS-CYP450*, and *TPS-BAHD* gene clusters may be coregulated upon attack by herbivores or to attract pollinators. Similar fluctuations of these transcripts grouped in one cluster were observed in our study (Fig. 6). For example, the expression levels of two clustered *TPS* genes (*La05G1453*/*La05G1454*) at Chr5 were largely increased in flowers treated with methyl jasmonate (Fig. 6b), and the other gene cluster, *La02G01528*/*La02G01529*/*La02G01630*, showed co-upregulation in JAL and elevated expression levels before blossoming (Fig. 6d). Overall, the gene-to-terpenoid network identifies several credible candidate genes involved in terpenoid accumulation.

## Discussion

Species evolution and diversity are often a result of genome duplication and the possible adaptive advantages it provides^22,35^. Our analyses, based on the whole-genome sequence of lavender, revealed that lavender experienced two additional lineage-specific WGDs since the γ triplication event shared by all eudicots. *Lavandula* and *Salvia* belong to the species-rich and chemically diverse subclade Nepetoideae within Lamiaceae. Among the mint subfamilies, Nepetoideae is the largest, as it contains almost half of the genera (~105/236) and approximately half of the species (~3600/7173) in the family. Within this genus, we found that the genome of *Sspl* underwent additional WGD events, similar to lavender, while a lineage-specific duplication event occurred in the evolutionary history of *Smil*. Neither *Sbai* from Scutellarioideae nor *Tgra* from Tectonoideae underwent an extra WGD after the γ event. Similarly, a study based on transcriptome data indicated that high levels of gene duplication events occurred in species-abundant Nepetoideae relative to the other subclades of Lamiaceae^17,28^. Most large angiosperm families, such as Brassicaceae, Fabaceae, Asteraceae, Poaceae, and Orchidaceae, have shown strong evidence of multiple polyploidization events in their evolutionary histories^36–38^. Therefore, ancient polyploidy has potentially contributed to the species richness of this large subclade of Lamiaceae^35^.

Lavender occurs naturally in the Mediterranean region. In addition to the γ triplication event shared by all eudicots, lavender underwent two lineage-specific rounds of WGD during the Eocene–Oligocene (~33.7 MYA) and Miocene–Pliocene (~5.3 MYA) transition. The penultimate WGD predicted in lavender (29.6 MYA) was dated close to the early Oligocene (33.5 MYA), when the Grande Coupure occurred in Europe with an abrupt cooling of the climate^39,40^. Moreover, the Mediterranean Sea became mostly desiccated owing to evaporation at approximately 5.6 MYA during the Messinian salinity crisis, which was close to the time of the latest WGD (6.9 MYA) in *Lang*. Previous reports of WGDs in various plant families have revealed the cooccurrence of polyploidy events and global climate changes^35,38^. Our results provide evidence supporting the global environmental selection of ancestral polyploids from different lineages. Organisms that underwent and survived WGD might gain some adaptive advantages, and the sudden gene increase may have enabled lavender to better cope with the dramatically changed environment. For instance, retentions following WGDs around the K-Pg (Cretaceous–Paleocene) boundary were commonly enriched for genes involved in the responses to low temperature and darkness^41^. In lavender, several expanded gene families were associated with terpenoid metabolism, plant hormone signal transduction, and plant–pathogen interactions via WGDs or tandem repeats. The high retention of *Bet v1*, *SAUR*, and terpenoid-related genes following duplication may deliver evolutionary advantages that allow lavender to survive under pathogen attack or abiotic stress^42,43^.

Previous studies have demonstrated that the patterns of gene retention following WGD are not random and become biased towards genes encoding proteins that play a role in gene networks and signaling cascades^44^. It has been reported that WGD probably contributes to specific trait innovations, particularly novelty in specialized metabolites^36,45^. Analyses of the whole genomes of *Tripterygium wilfordii* and *Litsea* suggest that duplicated gene families involved in terpenoid biosynthesis were associated with the production of their specific compounds^46,47^. Terpenoid metabolism could be further affected and favored by polyploidization, perhaps as an adaptive response. The present analyses of the copies and expression profiles related to the genes involved in terpenoid biosynthesis, such as those encoding the key rate-limiting enzymes DXS and TPS-b, suggest that the duplications of these gene families contributed to the volume and diversity of volatile terpenoid production in the GTs of lavender. Moreover, we also identified some *TPS* groups that occurred as local gene duplications and *TPS*-*BAHD*/*TPS*-*CYP450* gene clusters, which were prepared for abundant materials for gene functional differentiation and gene regulation divergence and have been described as important drivers of terpenoid diversity in plants^4,23^. As most terpenoid production is controlled primarily at the level of transcription^48^, the gene-to-terpenoid networks uncovered in our study through the integration of genomic, transcriptomic, and metabolomic data provide abundant promising candidates for terpenoid biosynthesis. Moreover, the genes that correlated with volatiles serving as major attractants and repellents were found to be distinctly separated, thus paving the way for future research regarding the interaction between insects and plant volatiles.

This study presents compelling evidence revealing widespread ancient polyploidy in Lamiaceae. *Lavandula* is an ecologically, economically, and culturally important genus, representing an excellent system for evolution-based studies of terpenoid metabolism. We produced a chromosome-level lavender reference genome using the latest sequencing technologies and bioinformatic methods, investigated the duplicates resulting from the two last WGDs and tandem repeats, and identified the candidate genes closely correlated to the primary attractive and defensive compounds. The expansion of gene families related to terpenoid metabolism, plant hormone signal transduction, and plant–pathogen interactions seemed to account for the increased diversity of volatile terpenoids and enhanced resistance to pathogen attack or abiotic stress. In summary, our data provides a stronger understanding of the evolution and diversification of Lamiaceae. Genetic evidence from the lavender genome sheds new light on the contribution of gene duplication to lavender adaptation. Moreover, the identification of candidate genes involved in the production of volatiles will dramatically improve the efficiency of genome editing to improve economic traits.

## Materials and methods

### Genome and transcriptome sequencing

The fresh leaves from which DNA was extracted for the genome sequencing of *L. angustifolia* ‘Jingxun 2’ were obtained from a single plant maintained at the Institute of Botany, Chinese Academy of Science. Four sequencing strategies were used to sequence the genome in our study. (i) Second-generation sequencing: A short paired-end Illumina DNA library with 350 bp insertions was sequenced on an Illumina NovaSeq 6000 (Illumina Inc, CA, USA) sequencer. We obtained 59.06 Gb clean data, which provided ~54× genome coverage. The abundance of 17 nt k-mers was used for genome survey analysis. (ii) Third-generation sequencing: according to the PacBio SMRT protocol, 50 µg of high molecular-weight genomic DNA was prepared to generate five standard SMRTbell libraries with a 20 kb insert size, and the resulting PacBio long reads were subsequently sequenced on the PacBio Sequel System (Pacific Biosciences, CA, USA). (iii) 10× Genomics library construction: a 10× Genomics DNA library was prepared following the manufacturer’s protocol (Chromium Genome v1, PN-120229). Sequencing was performed using the Illumina HiSeq 2500, and a total of 117.77 Gb clean data were obtained. (iv) Hi-C library construction: tissue culture seedlings propagated from the same plant were used as starting material for the Hi-C library. Several processes were required prior to DNA isolation: chromatin fixation with formaldehyde, digesting fixed chromatin with restriction enzymes, end-filling and biotin labeling, blunt end ligation with DNA ligase, releasing DNA molecules from crosslinks using protease, and shearing DNA into 350-bp fragments. Thereafter, the library was constructed using the fragments labeled with biotin and sequenced on the Illumina HiSeq PE150 instrument. In total, 135.11 Gb clean data were retained for further analysis.

Transcriptome data were generated with two sequencing approaches in our study. We collected RNA samples from different organs of lavender (glandular trichomes of the opening flower, root, stem, leaf, and flower at three different stages: flower bud, opening flower, and fading flower) when 70% of the flowers on spike opened within one plant. RNA from each tissue was extracted in triplicate. RNA samples of the glandular trichome, root, stem, and leaf were sequenced using the Illumina NovaSeq 6000 platform. We generated an average of 40.57 million paired-end reads for each sample. Equal concentrations of RNAs from different organs (root, stem, leaf, and flower at the three different stages mentioned earlier) were mixed, and a 20 kb SMRTbell Template library was prepared for sequencing on a PacBio Sequel instrument using the PacBio Iso-Seq protocol. Finally, we obtained a total of 11.35 Gb of full-length transcriptome data.

### Genome assembly and annotation

The lavender genome was de novo assembled based on PacBio long reads using FALCON. Errors in the PacBio reads were corrected within the FALCON pipeline. Contigs were first polished based on raw PacBio data and finally corrected using Illumina short reads with Pilon. Consensus sequences were further assembled with the assistance of clean data produced from the 10× Genomics library. Chromosome-scale assembly was generated using Hi-C scaffolding. Annotation of repeat elements, gene structure gene function, and non-coding RNA are detailed in Supplementary Method 2. Three methods were used to predict protein-coding genes: homology-based, de novo, and transcriptome-based.

### Genome evolution

OrthoMCL (http://orthomcl.org/orthomcl/) was used to construct orthologous gene families between *L. angustifolia* and 13 other plant species. MUSCLE was utilized to construct multiple sequence alignments of 59 single-copy orthologs among 13 species. RAxML software (version 7.2.3) was used to construct the maximum likelihood tree with the PROTGAMMAAUTO model by employing sequence alignments with *Vitis vinifera* (*Vvin*), *Arabidopsis thaliana* (*Atha*), *Populus trichocarpa* (*Ptri*), and *Rosa chinensis* (*Rchi*) as outgroups. The MCMCTree program of PAML (http://abacus.gene.ucl.ac.uk/software/paml.html) was applied to estimate divergence time using CDS alignments transformed from protein alignments. Four calibration values were selected from the TimeTree website (http://www.timetree.org). Expansion and contraction of the orthologous gene families were determined using CAFÉ 2.2 (Computational Analysis of gene Family Evolution). For WGD analysis, the syntenic regions between and within *L. angustifolia* (*Lang*), *Salvia miltiorrhiza* (*Smil*), *Scutellaria baicalensis* (*Sbai*), *Tectona grandis* (*Tgra*), *Salvia splendens* (*Sspl*), and *Vvin* were found by MCscanX based on all-to-all BLASTP results. The protein sequences of homologous gene pairs in the syntenic region were extracted and aligned using MUSCLE. Subsequently, protein sequence alignments were converted into CDS files, and 4DTv values were calculated based on the CDS alignments with HKY model correction. For identification of tandem duplications, the annotated protein sequences in the genomes of lavender and four other species (*Smil*, *Sspl*, *Sbai*, and *Tgra*) in Lamiaceae were compared using BLASTP, and the gene pairs with identity ≥ 50% and e-value ≤ 1e-20 were retained. Gene pairs that were continuously distributed in the genome and had no other genes interspersed between them were defined as candidate tandem gene duplications and then further confirmed this designation by gene function annotation.

### Genes related to terpenoid biosynthesis

Protein sequences annotated as involved in the terpenoid backbone biosynthesis pathway [including ACAT, HMGS, HMGR, MVK, PMK, MVD, DXR, DXS, MCT, CMK, MDS, HDS, HDR, IDI, and generate geranyl diphosphate synthetase (GPPS), farnesyl diphosphate synthetase ((FPPS)/GGPPPS/FPPS) in the Kyoto Encyclopedia of Genes and Genomes (KEGG) database (Ko00900) were retrieved from *Atha*, *Solanum lycopersicum* (*Slyc*) and *Nicotiana tabacum*. Then, we searched for homologs to these proteins in the genomes of lavender and eight other plant species, including *Atha*, *Helianthus annuus* (*Hann*), *Rchi*, *Slyc*, *Smil*, *Sspl*, *Sbai*, and *Tgra*, using BLASTP with an E-value cutoff of 1e−5. We predicted the *TPS* genes by both conserved-domain-based (PF01397 and PF03936) and homolog-based BLAST. Conserved domains were used as search queries against the predicted proteome using hmmsearch in HMMER. TPS protein sequences from *Atha*, *Vvin*, *Ptri*, and rice were used as queries to identify the TPSs of lavender, *Hann*, *Rchi*, *Slyc*, *Smil*, *Sspl*, *Sbai*, and *Tgra*. For BAHD identification, members of the BAHD family from *Atha* were used as queries to predict lavender BAHD using BLASTP (1e−5). To identify the *CYP450* genes, we used the CYP450 protein sequences of rice, *Atha*, and *Chlamydomonas reinhardtii* (http://drnelson.uthsc.edu/P450seqs.dbs.html) as queries to search for homologs and conserved domains (PF00067). The predicted CYP450 candidates were further grouped into clans and families, as previously described^20^.

### Solid-phase microextraction (SPME) coupled to GC-MS analysis

Fresh flowers (10 mg), fresh leaves (150 mg), and fresh stems (200 mg) were placed into headspace vials and kept in a laboratory water bath at 40 °C (for flower samples) or 70 °C (for leaf or stem samples) for 40 min. Twelve microliters of 3-octanol (Sigma Aldrich, Saint Louis, MO, USA) was added as an internal standard. SPME analysis (20 min exposure to a 2 cm DVB/CAR/PDMS fiber, Supelco, Bellefonte, PA, USA, followed by analyte desorption at 220 °C for 3 min) was performed using a Varian CP-3800/Saturn 2000 apparatus (Varian, Walnut Creek, CA, USA) equipped with a Zebron ZB-5 MSI (30 m × 0.25 mm × 0.25 µm) column (Phenomenex, Shim-Pol, Poland). The GC oven temperature was programmed from 50 °C to 130 °C at a rate of 4 °C/min; to 180 °C at a rate of 10 °C/min; and then to 280 °C at a rate of 20 °C/min. Scanning was performed from 35 to 550 *m*/*z* in electronic impact (EI) mode at 70 eV. Samples were injected in a split ratio of 80:1, and helium gas was used as the carrier gas at a flow rate of 1 ml/min. All analyses were run in triplicate.

### qRT-PCR analysis

The same RNA samples that were used in the microarray experiments, including various tissues (LAR, LAS, LAL, LAF, and LAGT) and flowers at different developmental stages (FB0, FB1, FB2, F3, F4, and F5), were used for qRT-PCR. FB0, FB1, and FB2 represent unopened flowers, F3 represents opening flowers, and F4 and F5 represent fading flowers. For different treatments, flowers (JAF) and leaves (JAL) were collected after treatment with 8 mM methyl jasmonate for 12 h. The control flowers and leaves are indicated as CKF and CKL. The experimental methodology and qRT-PCR analysis were performed according to Li et al.^48^. The primer sequences used in our study are listed in Supplementary Table S27.

## Supplementary information

Supplemental Information

Table S1. Sequencing data output statistics of genome survey

Table S2. Statistics of the genomic characteristics of lavender obtained by genome survey analysis based on k-mer=17

Table S3. Statistics on sequencing of the lavender genome

Table S4. Statistics of reads coverage in lavender genome

Table S5. Lavender genome CEGMA assessment results

Table S6. Lavender genome BUSCO assessment results

Table S7. The Hi-C construction library quality assessment

Table S8. Lavender chromosome length distribution results

Table S9. Statistics on base content of lavender genome

Table S10. The summary of data quality for five different tissues of RNA-Seq in lavender

Table S11. The summary of data quality for a mixed lavender RNA sample of SMRT-Seq

Table S12. Statistics of repeat sequence in lavender genome

Table S13. Statistics of transposable element classification in lavender genome

Table S14. Basic statistical results of gene structure prediction

Table S15. Statistical results of gene functional annotations

Table S16. Basic statistical results of the genetic structure of L. angustifolia and other eight representative species

Table S17. Statistics of non-coding RNA in lavender genome

Table S18. KEGG pathway enrichment of gene retained in the penultimate WGD event

Table S19. KEGG pathway enrichment of gene retained in the lasted WGD event

Table S20. Tandem duplications identified in lavender genome

Table S21. Tandem duplications identified in Sbai, Smil, Sspl, Tgra and Sind genome

Table S22. Analysis of volatile terpenoids identified by GC-MS

Table S23. Copy number variation of genes involved in the terpenoid precursor biosynthesis in the 11 plant species

Table S24. Copy number variation of genes encoding TPSs in the 11 plant species

Table S25. The genomic location of representative gene clusters

Table S26. The candidate genes involved in the gene-terpenoid networks

Table S27. Gene-specific primer pairs used for qRT-PCR

## Data Availability

The raw genome and transcriptome sequencing data reported in this paper have been deposited in the National Center for Biotechnology Information (NCBI) database under project number PRJNA642976.
