# Correction: The chromosome-based lavender genome provides new insights into Lamiaceae evolution and terpenoid biosynthesis

**DOI:** 10.1038/s41438-021-00536-9

**Published:** 2021-04-15

**Authors:** Jingrui Li, Yiming Wang, Yanmei Dong, Wenying Zhang, Di Wang, Hongtong Bai, Kui Li, Hui Li, Lei Shi

**Affiliations:** 1 Key Laboratory of Plant Resources and Beijing Botanical Garden, Institute of Botany, Chinese Academy of Sciences, Xiangshan, 100093 Beijing, China; 2 University of Chinese Academy of Sciences, 100015 Beijing, China; 3 Novogene Bioinformatics Institute, 100083 Beijing, China

**Keywords:** Genome, Plant evolution

Correction to: *Horticulture Research*

10.1038/s41438-021-00490-6 published online 01 March 2021

After the publication of this article^1^, the authors became aware that the legends of Figs. 3 and 4 were inverted. The correct version is shown below.

**Fig. 3:**
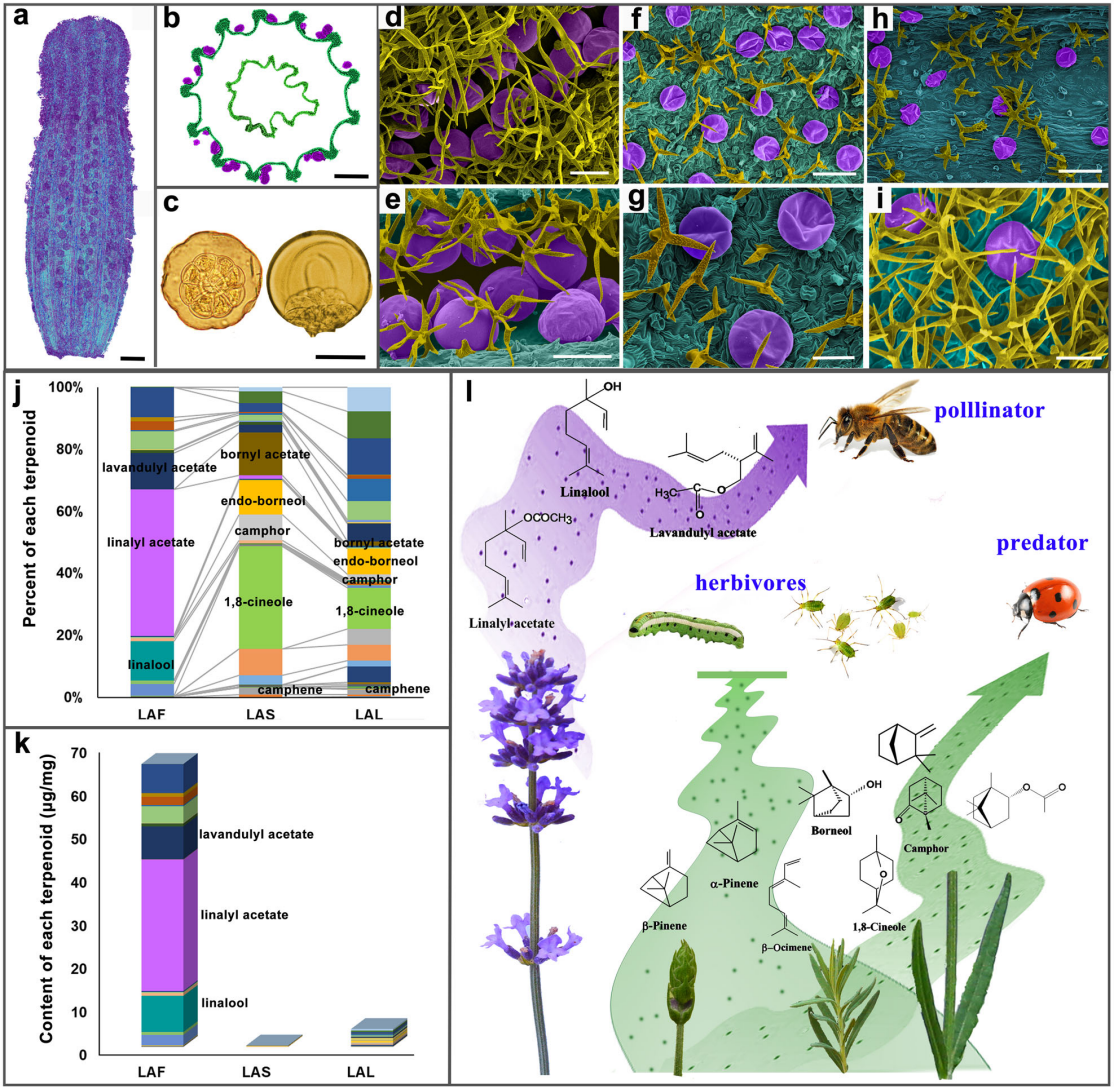
The sites, types, contents, and putative functions of volatile production in lavender. **a**, **b** Surface and cross-section of calyx of a blossom floret. These images were captured by CT. The glandular trichomes (GTs) of lavender are colored purple. **c** Top view and side view of a single GT separated from a flower at blossom. The GTs are composed of eight secretory cells and one secretory cavity. **d**–**i** Scanning electron microscopy images. The GTs of the flower (LAF), leaf (LAL), and stem (LAS) are colored purple, and non-GTs are in yellow. Scale bars = 1 mm (**a**, **b**); 50 μm (**c**–**e**, **g**, **i**); and 100 μm (**f**, **h**). **j**, **k** The relative and absolute contents of volatile terpenoids in LAF, LAL, and LAS. **l** The ecological function of the main volatiles emitted by opening flowers, flower buds, leaves, and stems. A large proportion of linalool, linalyl acetate, and lavandulyl acetate in opening flowers function as attractants for pollinators. At the flower bud stage, *α*-pinene, *β*-pinene, and *β*-ocimene, etc. provide defense against herbivores and predators. Borneol, camphor, 1,8-cineole, camphene, and bornyl acetate are the main compounds in leaves and stems, and are always repellents to pests

**Fig. 4:**
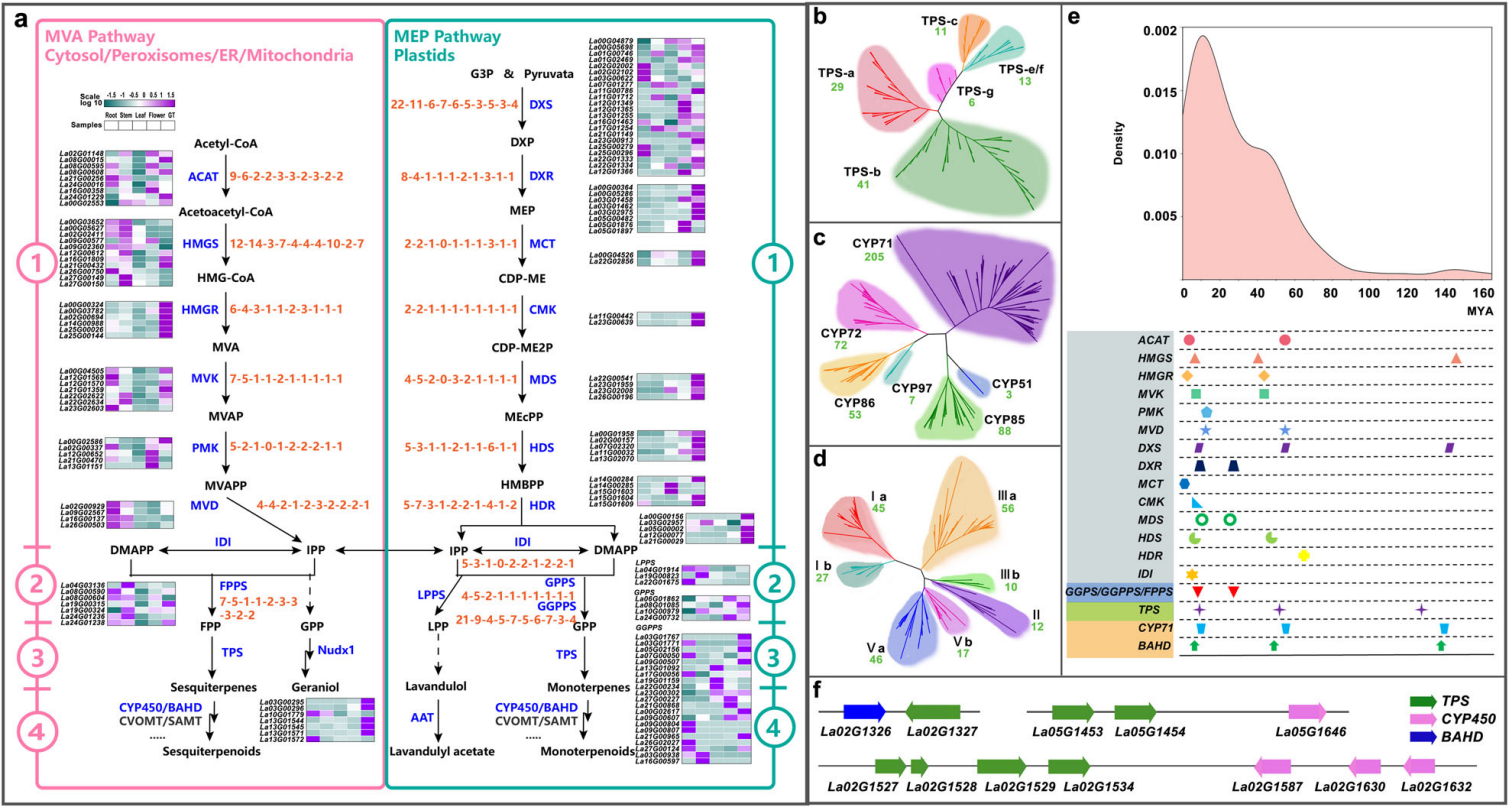
Biosynthesis of volatile terpenoids in lavender. **a** There are four steps required to produce diverse terpenoids. Enzymes involved at each step of the volatile terpenoid biosynthesis pathway are shown in blue, and intermediates are shown in black. Relative expression profiles of genes implicated in volatile terpenoid biosynthesis among various tissues (LAR, root; LAS, stem; LAL, leaf; LAF, flower; LAGT, glandular trichome) are presented as heatmaps (cyan–purple scale). Copy number variations of genes involved in volatile terpenoid biosynthesis in the ten plant species (from left to right: *Lang*, *Sspl*, *Tgra*, *Smil*, *Sbai*, *Sind*, *Slyc*, *Hann*, *Rchi* and *Atha*) are shown in orange font. **b**–**d** Phylogeny of TPS subfamilies (**b**), CYP450 clans (**c**), and BAHD subfamilies (**d**) in lavender based on protein sequences. The gene numbers clustered into one category are indicated in green font. **e** Ks values and duplication/divergence times of genes involved in terpenoid biosynthesis in lavender. **f** Representative gene cluster with physical link. Clusters *TPS-TPS*, *TPS-BAHD*, and *TPS-CYP450* are shown

In addition, the *Lavandula angustifolia* ‘Jingxun 2’ was mistakenly edited as *Lavandula angustifolia* “Jingxun 2”.

The authors would like to apologize for above error.

The original article has been corrected.
