# Supplementary material for: The chromosome-based lavender genome provides new insights into Lamiaceae evolution and terpenoid biosynthesis
Source: Hortic Res. 2021 Mar 1;8:53. doi: 10.1038/s41438-021-00490-6 (PMC7917091; doi:10.1038/s41438-021-00490-6)
Supplement: Supplementary file 1 — Supplemental Information [file 41438_2021_490_MOESM1_ESM.docx]

Supplemental Information

**Title:** The chromosome-based lavender genome provides new insights into Lamiaceae evolution and terpenoid biosynthesis

**Authors:** Jingrui Li^#^, Yiming Wang^#^, Yanmei Dong, Wenying Zhang, Di Wang, Hongtong Bai, Kui Li*, Hui Li*, Lei Shi*

**Methods S1. Genome assembly.**

*De novo* genome assembly. Before assembling, the ‘daligner’ executed by the main script of the FALCON1 assembler was used to correct PacBio long reads error with the PacBio short reads less than 7000 bp to generate consensus sequences. After error correction, the consensus sequences can achieve accuracies up to 99.999%. Then, FALCON identified the overlaps between all pairs of the preassembled error-corrected reads. The read overlaps were used to construct a directed string graph following the Myers algorithm. Contigs were constructed by finding the paths from the string graph (falcon_sense_option = -- output_multi --min_idt 0.70 --min_cov 4 --max_n_read 300 --n_core 8; overlap_filtering_setting = --max_diff 100 --max_cov 100 --min_cov 4 --n_core 12). The error correction of preceding assembly was performed by consensus–calling algorithm Quiver2 with Pacbio reads. The Illumina reads were also used to correcte the contigs with Pilon3. The error corrected contigs were then removed heterozygosity by purge_haplotigs with Illumina clean reads. The fragScaff software was mainly used for 10× Genomics Scaffolds Extending. The procedures are as follows: 1) Linked-reads generated using the 10× Genomics library were aligned to the consensus sequence of PacBio assembly result to obtain the superScaffold using BOWTIEv2; 2) With the actual distance of consensus sequence increased, the Linked-reads that support its connection will reduce. The consensus sequence without the linked-reads support will be filtered and only the consensus sequence with the linked-reads support will be used for the subsequent assembly. FragScaff uses parameters ‘ -fs1 '-m 3000 -q 30 -E 30000 -o 60000' -fs2 '-C 3' -fs3 '-j 1 -u 3' ’. These processes yielded a final draft lavender genome assembly with a total length of 914.42 Mb, contig N50 of 1.196 Mb, and scaffold N50 of 2.01 Mb.

Hi-C technology assisted genome assembly. The Hi-C clean data was aligned to the preceding assembly using BWA software. Only the read pairs with both reads in the pair aligned to contigs are considered for scaffolding. For each read pair, its physical coverage is defined as the total bases spanned by the sequence of reads and the gap between the two reads when mapped to contigs. Per base physical coverage for each base in the contig is defined as the number of read pairs’ physical coverage it is part of. The misassembly can be detected by the sudden drop in per-base physical coverage in a contig. According to physical coverage of the alignment result, the mis-assemblies will be sheared to correct the misassemble errors by SALSA4. According to the linkage information and restriction enzyme site, the string graph formulation was used to construct the scaffold graph with LACHESIS5.

**Methods S2. Genome annotation**

Repeat annotation. A combined strategy based on homology alignment and *de novo* search to identify the whole genome repeats were applied in our repeat annotation pipeline. Tandem Repeat was extracted using TRF (http://tandem.bu.edu/trf/trf.html) by *ab initio* prediction. The homolog prediction commonly used Repbase (<http://www.girinst.org/repbase>) database employing RepeatMasker (http://www.repeatmasker.org/) software and its in-house scripts (RepeatProteinMask) with default parameters to extracted repeat regions. And ab initio prediction built *de novo* repetitive elements database by LTR_FINDER (http://tlife.fudan.edu.cn/ltr_finder/), RepeatScout (http://www.repeatmasker.org/), RepeatModeler (http://www.repeatmasker.org/RepeatModeler.html) with default parameters, then all repeat sequences with lengths >100bp and gap ‘N’ less than 5% constituted the raw transposable element (TE) library. A custom library (a combination of Repbase and our *de novo* TE library which was processed by uclust to yield a non-redundant library) was supplied to RepeatMasker for DNA-level repeat identification.

Structure annotation. Structural annotation of the genome incorporates ab initio prediction, homology-based prediction and RNA-Seq assisted prediction, was used to annotate gene models. i) Homolog prediction. Sequences of homologous proteins were downloaded from Ensembl/NCBI/others. Protein sequences were aligned to the genome using TblastN (v2.2.26; E-value ≤ 1e-5), and then the matching proteins were aligned to the homologous genome sequences for accurate spliced alignments with GeneWise (v2.4.1) software which was used to predict gene structure contained in each protein region. ii) Ab initio- prediction. For gene predication based on Ab initio, Augustus (v3.2.3), Geneid (v1.4), Genescan (v1.0), GlimmerHMM (v3.04) and SNAP were used in our automated gene prediction pipeline. iii) Gene prediction by *RNA-seq* and full-length transcriptome. Transcriptome reads assemblies were generated with Trinity (v2.1.1) for the genome annotation. To optimize the genome annotation, the RNA-Seq reads from different tissues which were aligned to genome fasta using TopHat (v2.0.11) with default parameters to identify exons region and splice positions. The alignment results were then used as input for Cufflinks (v2.2.1) with default parameters for genome-based transcript assembly. The non-redundant reference gene set was generated by merging genes predicted by three methods with Evidence Modeler (EVM, v1.1.1) using PASA (Program to Assemble Spliced Alignment) terminal exon support and including masked transposable elements as input into gene prediction. Individual families of interest were selected for further manual curation by relevant experts.

Functional annotations. Gene functions were assigned according to the best match by aligning the protein sequences to the Swiss-Prot using Blastp (with a threshold of E-value ≤ 1e-5). The motifs and domains were annotated using InterProScan (v4.8) by searching against publicly available databases, including ProDom, PRINTS, Pfam, SMRT, PANTHER and PROSITE. The Gene Ontology (GO) IDs for each gene were assigned according to the corresponding InterPro entry. We predicted the proteins function by transferring annotations from the closest BLASThit (E-value <10-5) in the Swissprot database and BLAST hit (E-value <10-5) in the NRdatabase. We also mapped gene set to a KEGG pathway and identified the best match for each gene.

Non-coding RNA annotation. The tRNAs were predicted using the program tRNAscan-SE (*http://lowelab.ucsc.edu/tRNAscan-SE/*). For rRNAs are highly conserved, we choose relative species’ rRNA sequence as references, predict rRNA sequences using Blast. Other ncRNAs, including miRNAs, snRNAs were identified by searching against the Rfam database with default parameters using the infernal software ([*http://infernal.janelia.org/*](http://infernal.janelia.org/)).

**Supplementary figures**


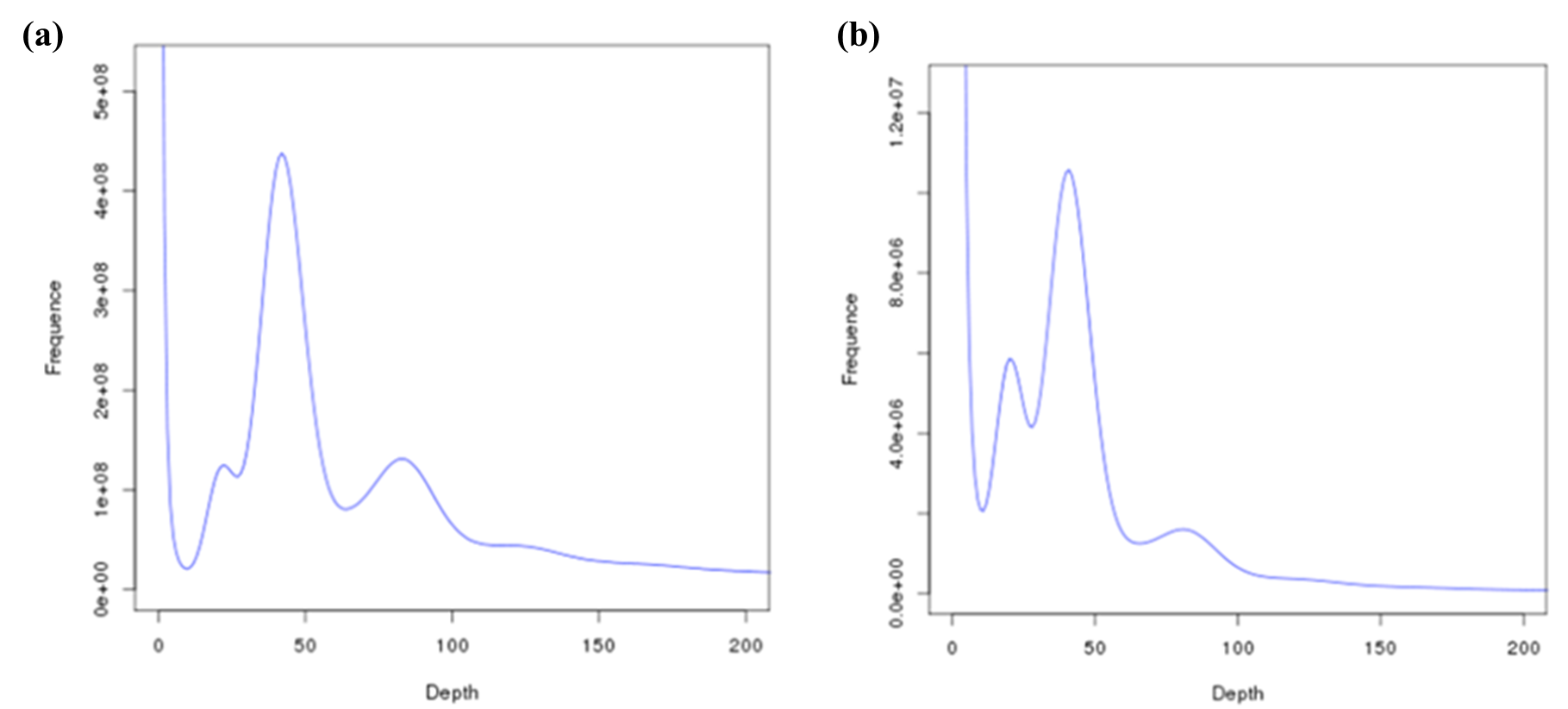


**Fig. S1** 17-mer analysis to estimate the *L. angustifolia* ‘Jingxun 2’ genome size. (a) K-mer = 17 Depth and K-mer number frequency distribution. (b) K-mer = 17 Depth and K-mer type frequency distribution.


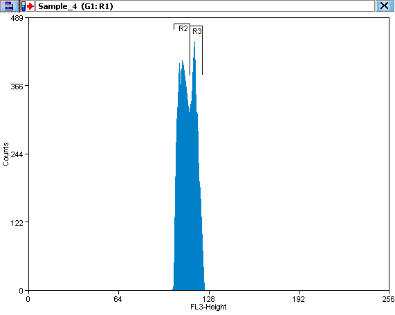


**Fig. S2** Estimation of the *L. angustifolia* ‘Jingxun 2’ genome size via 17-mer analysis. The genome size of *L. angustifolia* was estimated at 1016 Mb using *Solanum lycopersicum* (942 Mb) as control. R2 peak represents fluorescence intensity of *Solanum lycopersicum* , and R3 peak is fluorescence intensity of *L. angustifolia*.


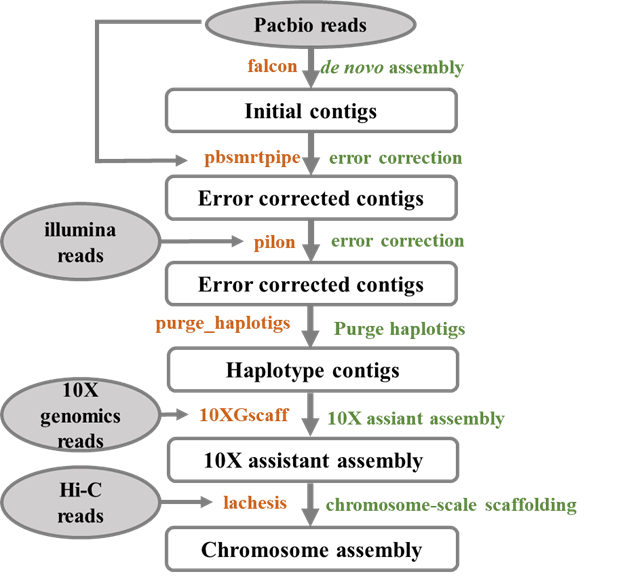


**Fig. S3** The lavender genome assembly workflow. The sequencing data used to assembly was indicated in gray circles. The orange fonts are indicative of software and the green fonts are processes in every step.


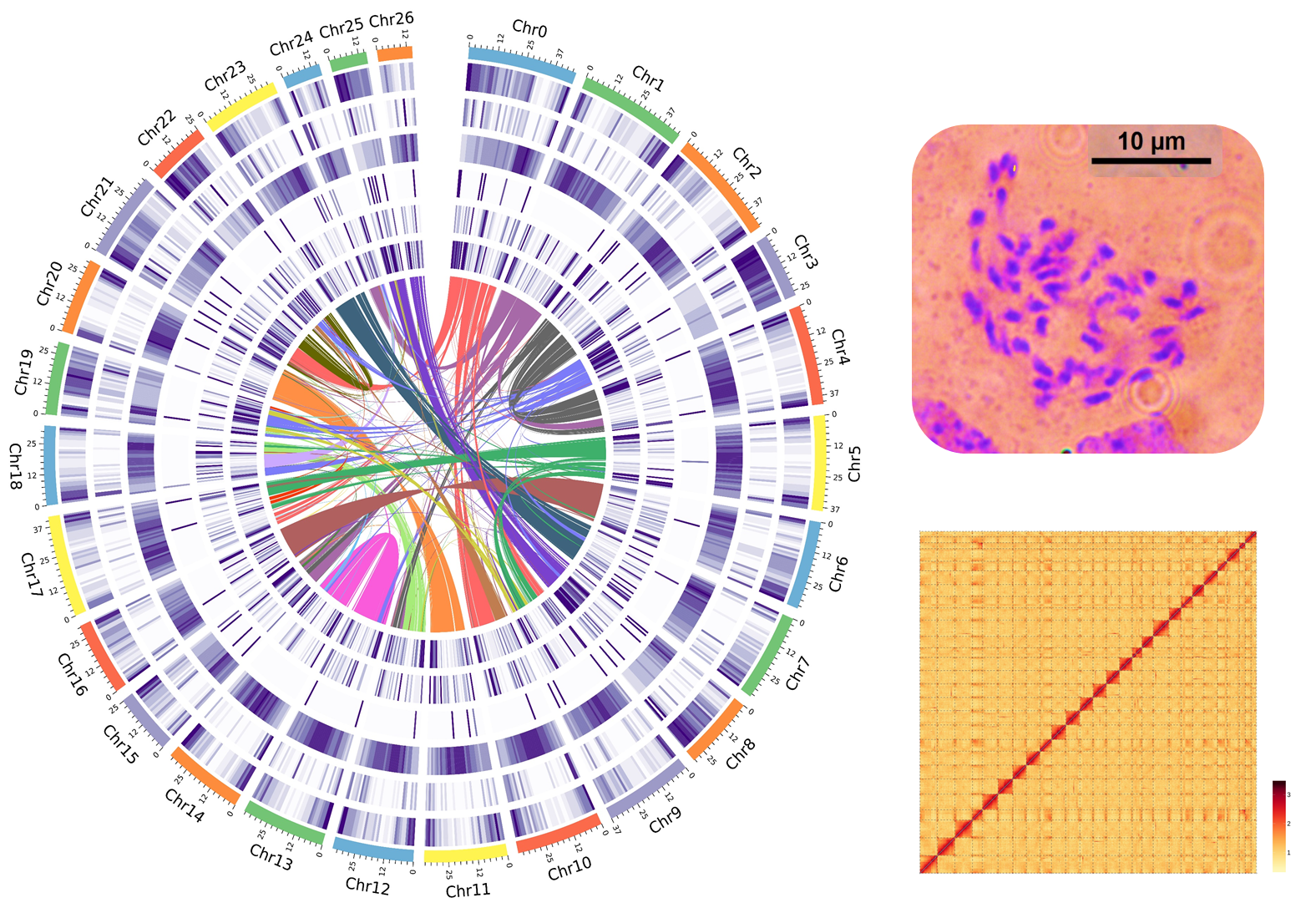


**Fig. S4** The karyotype of *L. angustifolia* ‘Jingxun 2’. The basic root-tip metaphase cells show the number of chromosomes number (2n = 54).


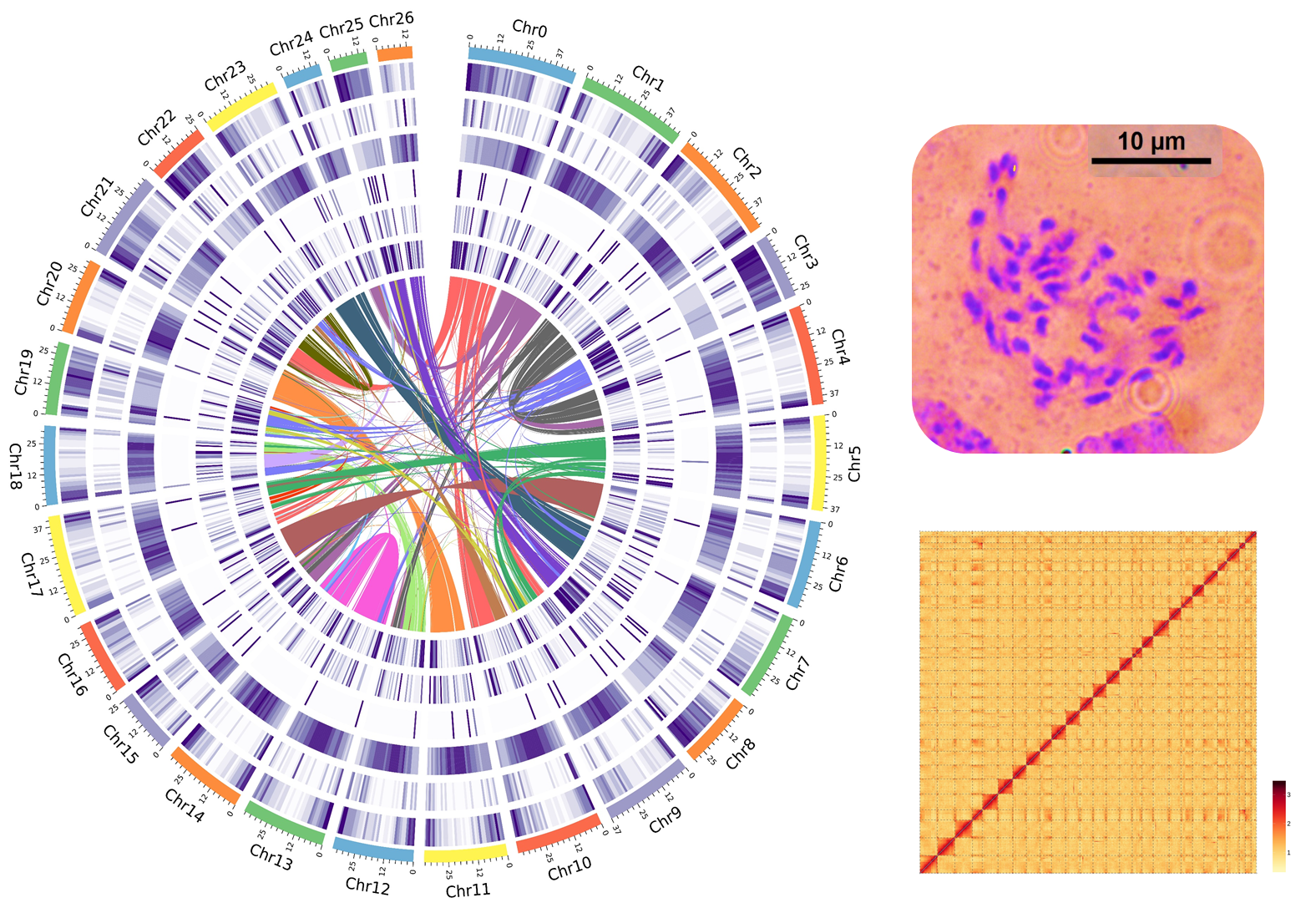


**Fig. S5** Interchromosomal Hi-C contact map of lavender. The intensity of each pixel represents the number of Hi-C links of 500 kb resolution in the chromosomes. Darker red pixels denote higher contact probabilities. Most interactions were observed within the chromosomes.

**
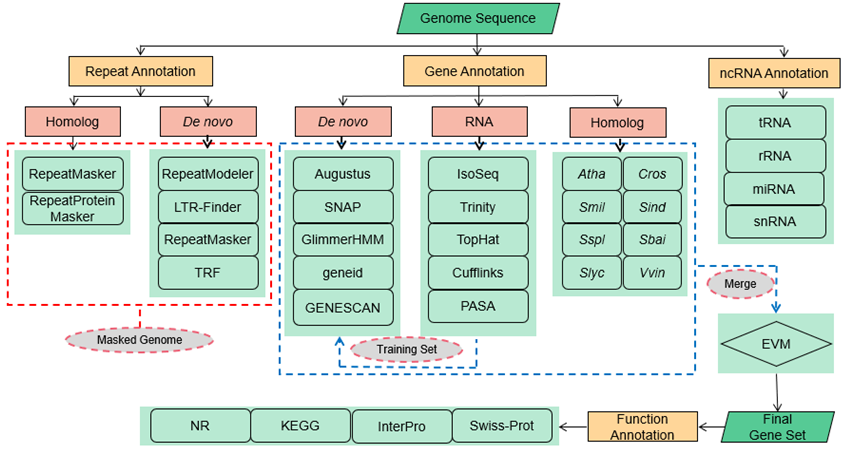
**

**Fig. S6** Diagrammatic sketch of the annotation pipeline.

**
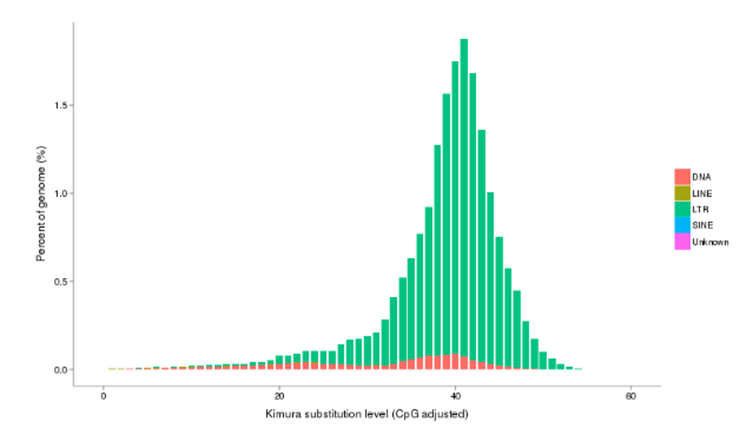
**

**Fig. S7** Transposable element sequence bifurcation distribution.


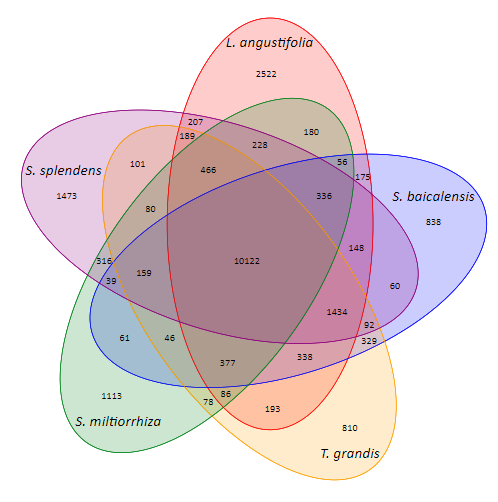


**Fig. S8** Statistical results of gene families in lavender and other species of Lamiaceae.


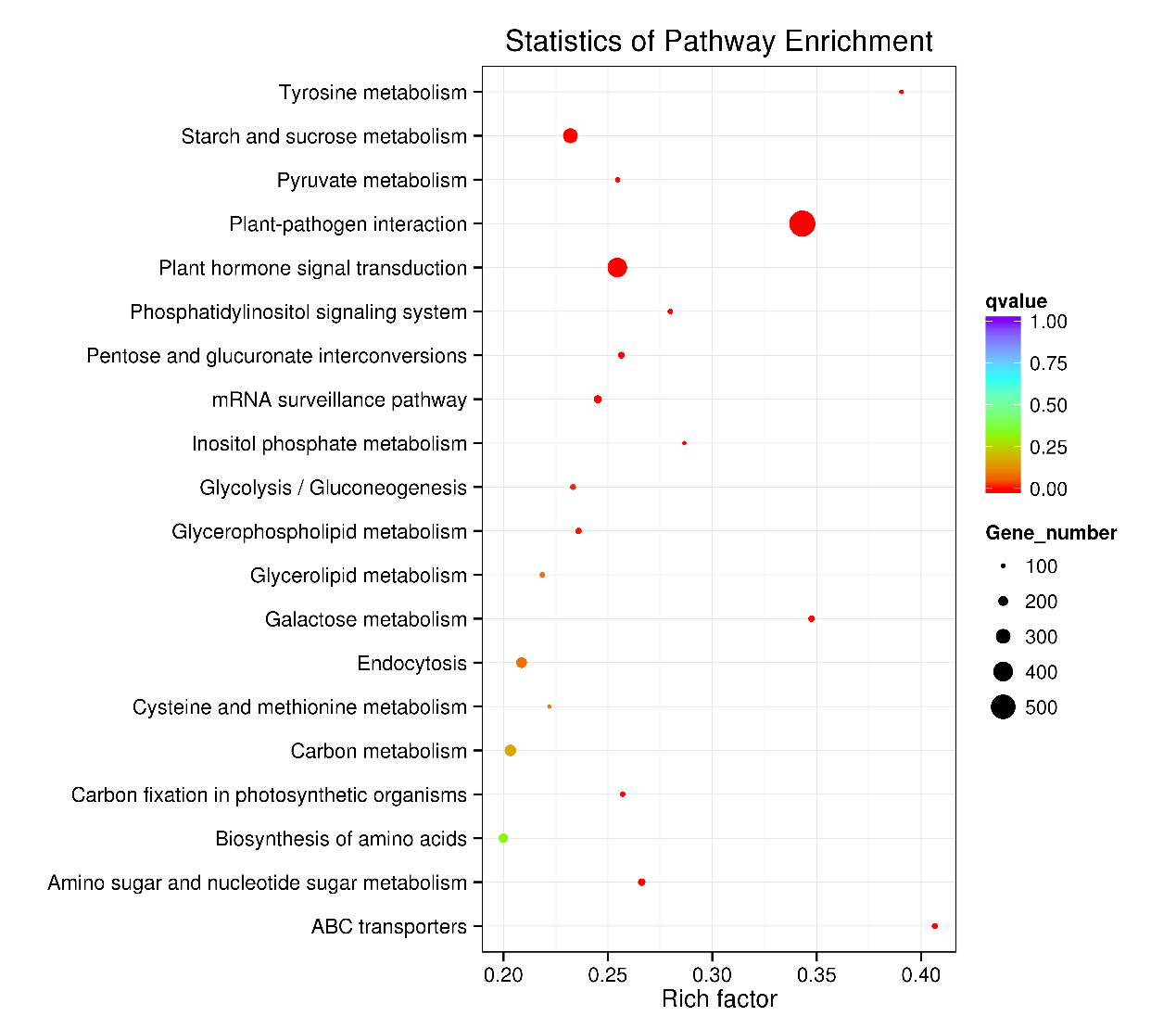


**Fig. S9** KEGG pathway enrichment of expanded genes in lavender.


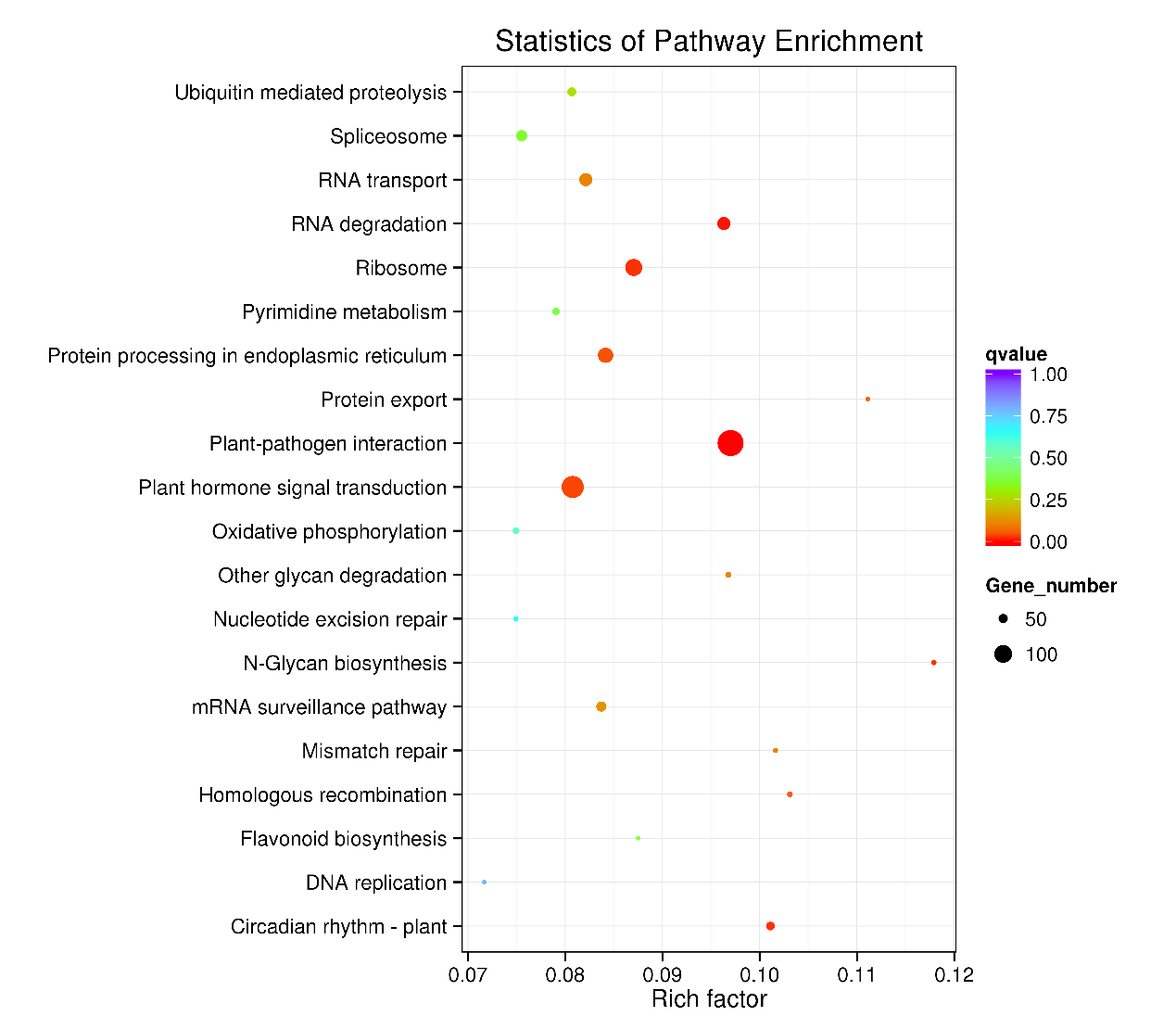


**Fig. S10** KEGG pathway enrichment of genes specific to lavender.


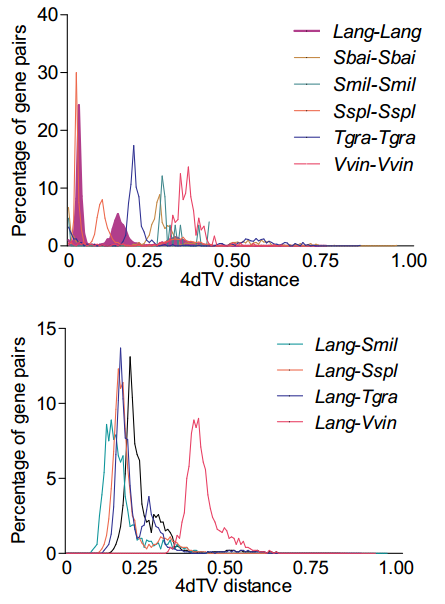


**Fig. S11** Distribution of 4DTv shown in colored lines as indicated.


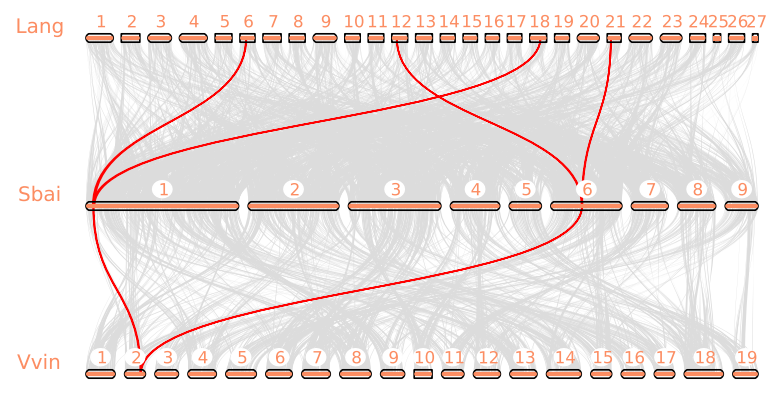


**Fig. S12** Microsynteny among *Vvin*, *Sbai* and *Lang*.


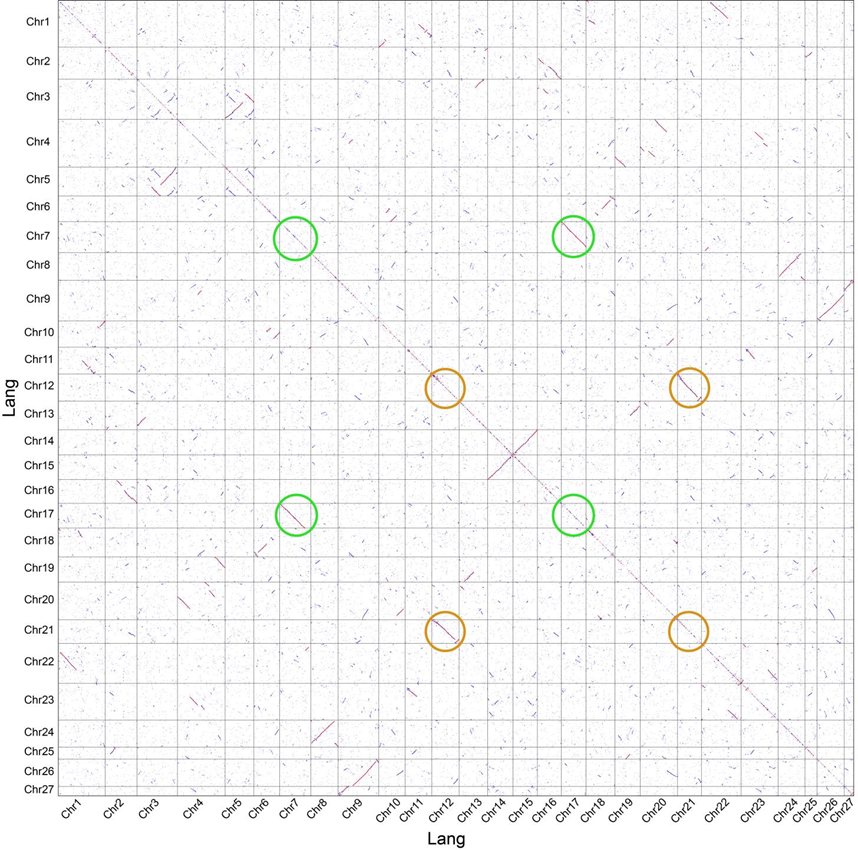


**Fig. S13** Dot plot of paralogs in the lavender genome. Green and orange circles are examples of 1:2 chromosomal relationships in lavender.


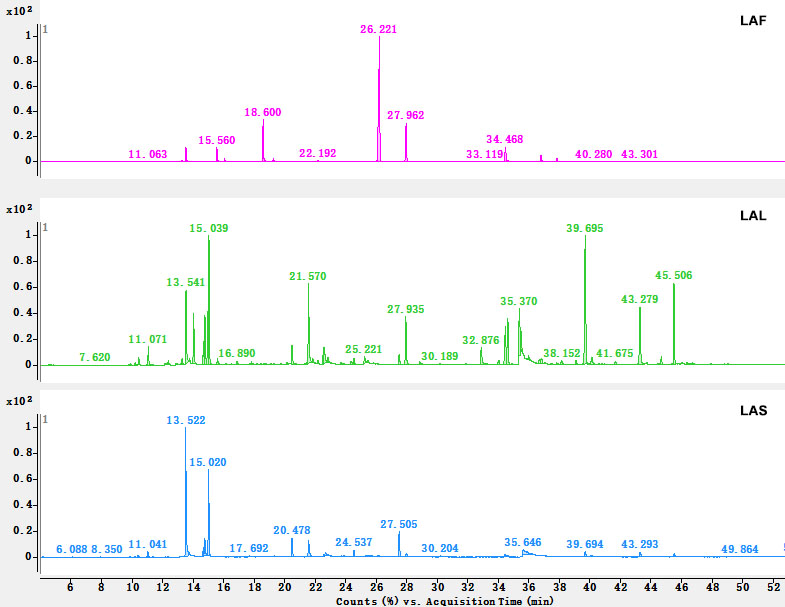


**Fig. S14** GC–MS total ion chromatograms of volatiles collected from lavender flowers (LAF), leaves (LAL) and stems (LAS).


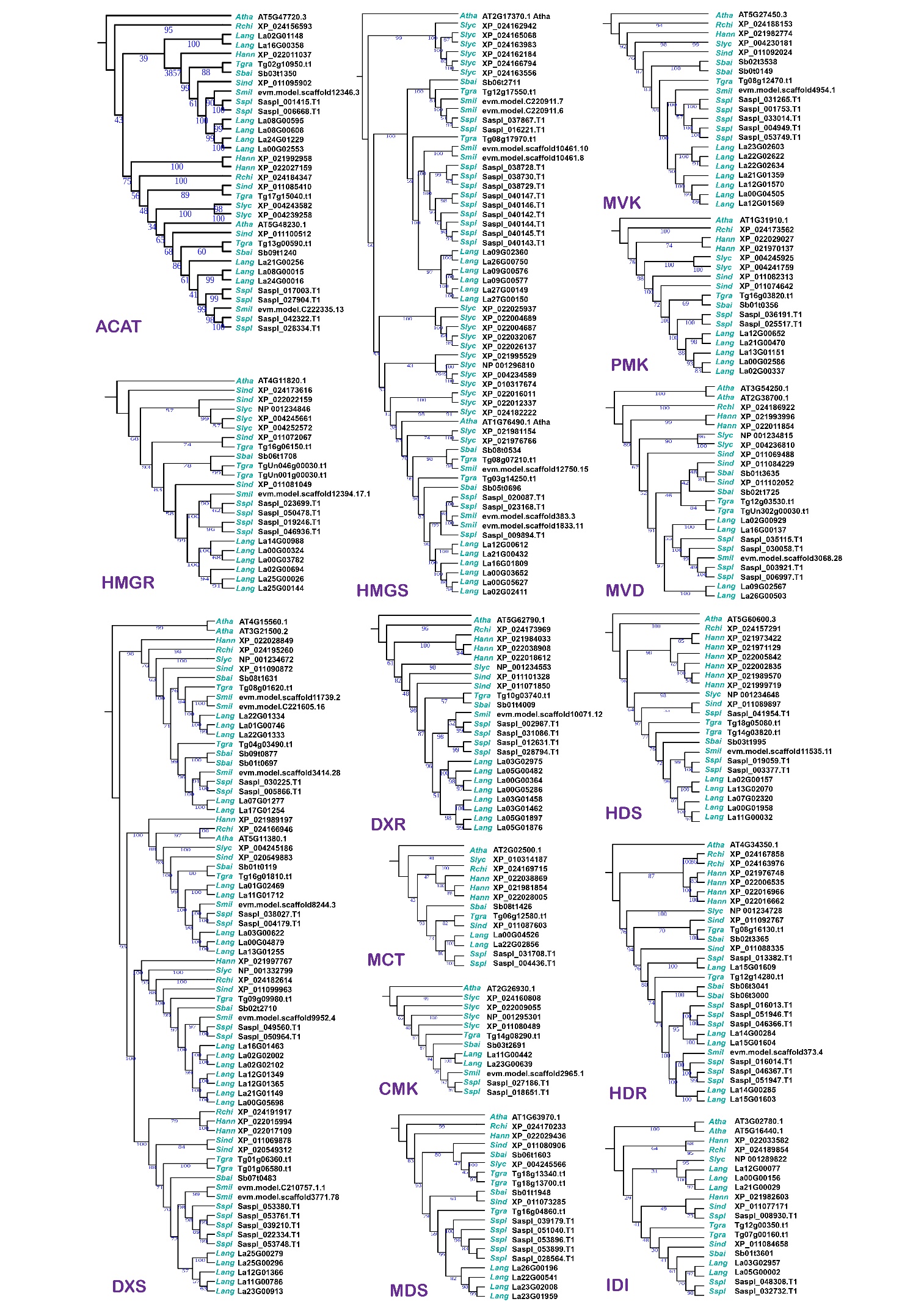


**Fig. S15** Phylogenetic trees of the genes involved in the MEP/MVA pathway. Each terpenoid biosynthetic gene family was construct phylogenetic trees using the maximum likelihood method. The blue fonts indicated the bootstrap values. The cyan nodes represent the species, including *Lang*, *Sspl*, *Smil*, *Sbai*, *Tgra,* *Sind*, *Slyc*, *Hann*, *Rchi*, and *Atha*.


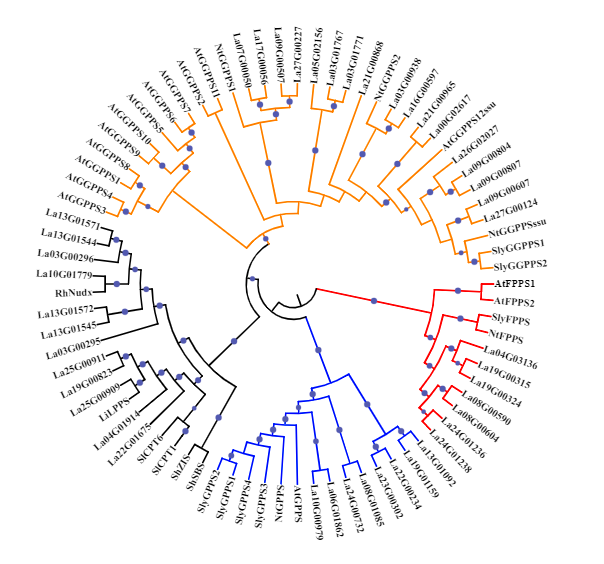


**Fig. S16** Phylogenetic trees of the genes involved in the prenyltransferase biosynthetic pathway. The Branches colored with red, blue and orange indicate members of FPPS, GPPS and GGPPS, respectively. And the black branches indicate otherprenyltransferases, such as LPPS and CPT.

**
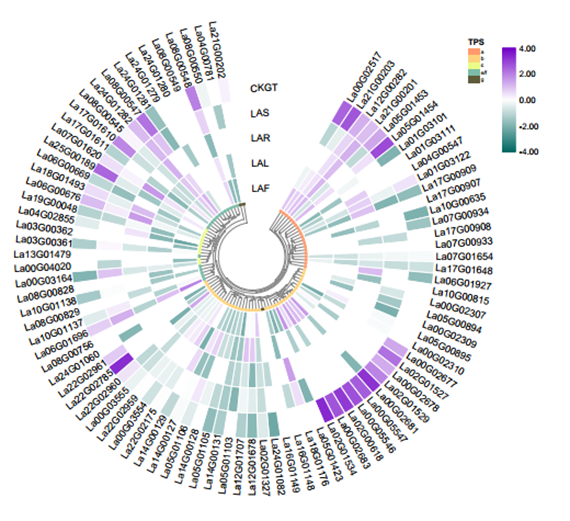
**

**Fig. S17** The expression of *TPSs*.


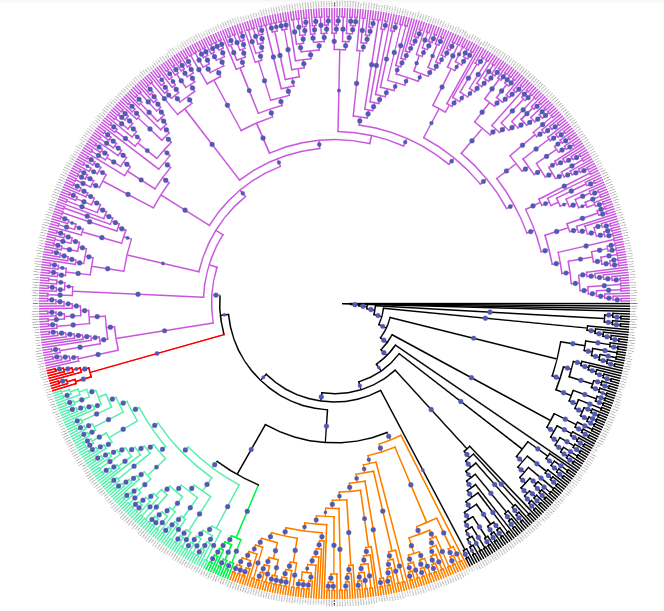


**Fig. S18** Phylogenetic tree of the CYP450 genes. Total CYP450 members of *L. angustifolia* and Arabidopsis were used to construct the phylogenetic tree using maximum likelihood method and Arabidopsis’ sequences as outgroup. The blue circles indicated the bootstrap values greater than 70. Branches colored with purple, red, cyan, green, orange, black indicate members of CYP71, CYP51, CYP86, CYP97, CYP72 and CYP85 clan, respectively.


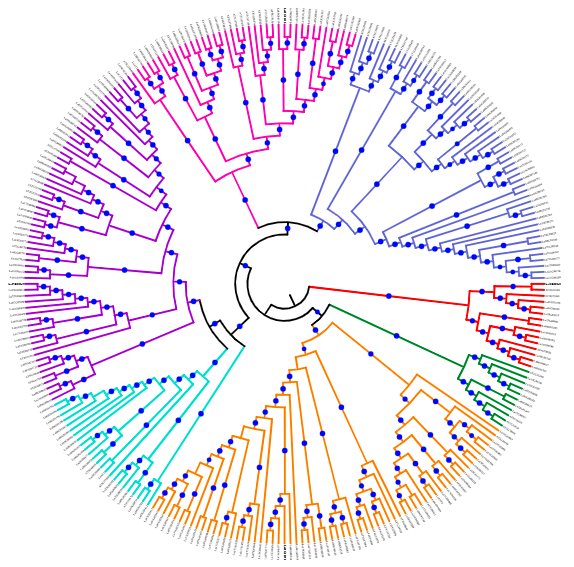


**Fig. S19** Phylogenetic tree of the BAHD genes. Total BAHD members from *L. angustifolia* and Arabidopsis were used to construct the phylogenetic tree using maximum likelihood method and Arabidopsis’ sequences as outgroup. The blue circles indicated the bootstrap values greater than 70. Branches colored with blue, magenta, red, orange, green, purple and cyan indicate members of BAHD Ⅰ, BAHD Ib, a BAHD Ⅱ, BAHD Ⅲ a, BAHD Ⅲ b, BAHD Ⅳ, BAHD Va and BAHD Vb subgroups, respectively.


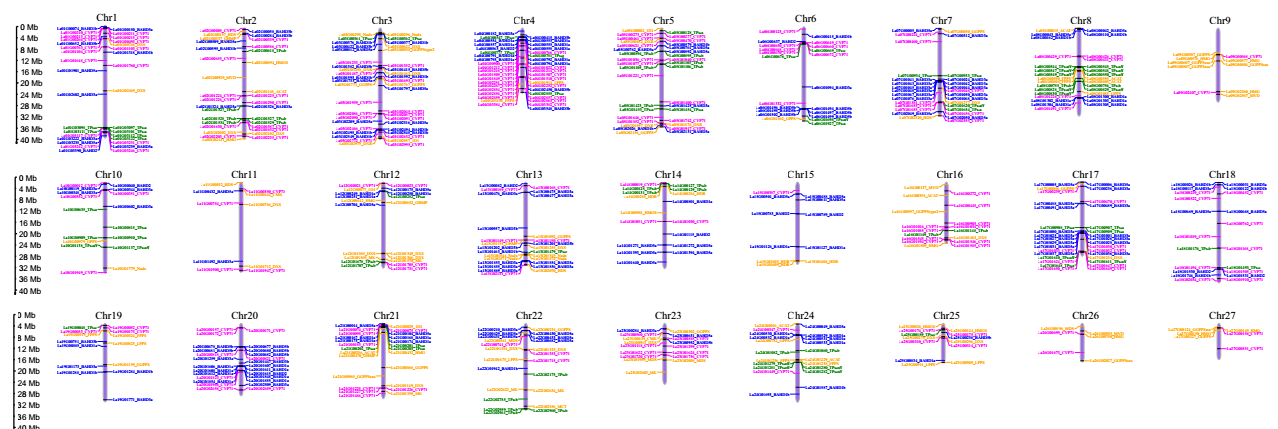


**Fig. S20** Chromosome location of terpenoid biosynthesis genes. Characters colored with green, orange, magenta, blue indicate genes involved in *TPSs*, MEP/MVA pathway, *CYP 71* clan and *BAHD* gene families, respectively.


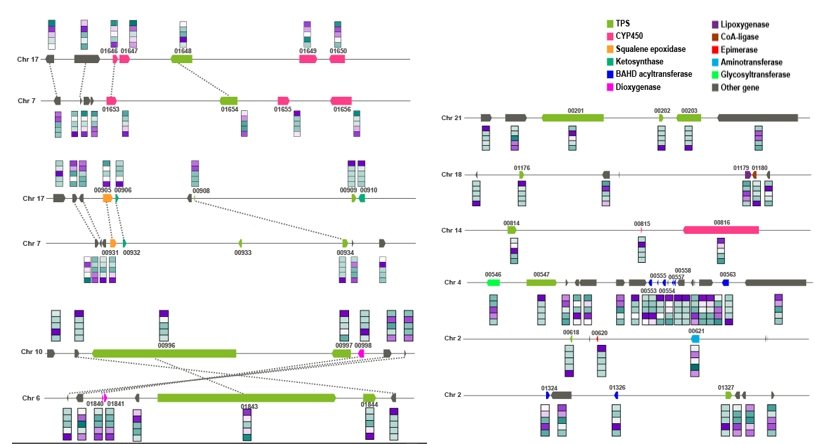


**Fig. S21** Gene clusters related to terpenoid metabolism predicted by plantiSMASH.


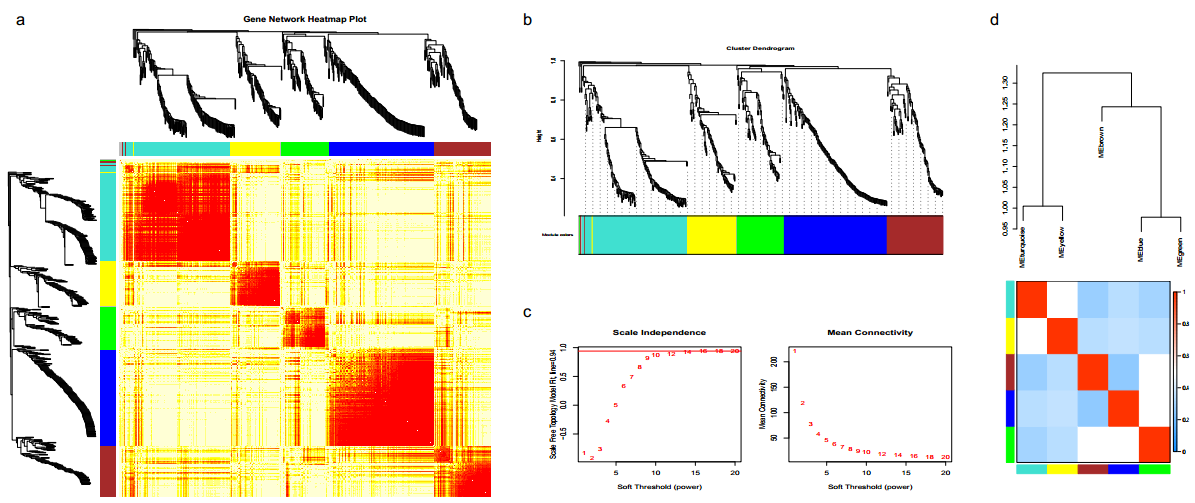


**Fig. S22** Clustering of gene expression profiles by WGCNA. (a) Expression heatmap of random genes clustered in five modules. (b) Hierarchical cluster tree showing five modules of co-expressed genes. Each of the gene is represented by a leaf of the tree and major tree branches constitute five modules, labeled with different colors at the lower panel. (c) The selection of soft threshold power. (d) The correlations between modules and modules.
